# Utilizing novel diversity estimators to quantify multiple dimensions of microbial biodiversity across domains

**DOI:** 10.1186/1471-2180-13-259

**Published:** 2013-11-15

**Authors:** Hannah M Doll, David W Armitage, Rebecca A Daly, Joanne B Emerson, Daniela S Aliaga Goltsman, Alexis P Yelton, Jennifer Kerekes, Mary K Firestone, Matthew D Potts

**Affiliations:** 1 Environmental Science, Policy, and Management, University of California, Berkeley, California 94720, USA; 2 Integrative Biology, University of California, Berkeley, California 94720, USA; 3 Plant and Microbial Biology, University of California, Berkeley, California 94720, USA; 4 Ecology Department, Earth Sciences Division, Lawrence Berkeley National Laboratory, Berkeley, California 94720, USA; 5 Current address: Department of Microbiology, The Ohio State University, Columbus, Ohio 43210, USA; 6 Earth and Planetary Science, University of California, Berkeley, California 94720, USA; 7 Current address: Cooperative Institute for Research in Environmental Sciences, University of Colorado, Boulder, Colorado 80309, USA; 8 Current address: Department of Microbiology and Immunology, School of Medicine, Stanford University, Stanford, California 94305, USA; 9 Current address: Civil and Environmental Engineering, Massachusetts Institute of Technology, Cambridge, Massachusetts 02139, USA

**Keywords:** Diversity indices, Diversity profiles, Phylogenetic diversity, Effective numbers, Community similarity

## Abstract

**Background:**

Microbial ecologists often employ methods from classical community ecology to analyze microbial community diversity. However, these methods have limitations because microbial communities differ from macro-organismal communities in key ways. This study sought to quantify microbial diversity using methods that are better suited for data spanning multiple domains of life and dimensions of diversity. Diversity profiles are one novel, promising way to analyze microbial datasets. Diversity profiles encompass many other indices, provide effective numbers of diversity (mathematical generalizations of previous indices that better convey the magnitude of differences in diversity), and can incorporate taxa similarity information. To explore whether these profiles change interpretations of microbial datasets, diversity profiles were calculated for four microbial datasets from different environments spanning all domains of life as well as viruses. Both similarity-based profiles that incorporated phylogenetic relatedness and naïve (not similarity-based) profiles were calculated. Simulated datasets were used to examine the robustness of diversity profiles to varying phylogenetic topology and community composition.

**Results:**

Diversity profiles provided insights into microbial datasets that were not detectable with classical univariate diversity metrics. For all datasets analyzed, there were key distinctions between calculations that incorporated phylogenetic diversity as a measure of taxa similarity and naïve calculations. The profiles also provided information about the effects of rare species on diversity calculations. Additionally, diversity profiles were used to examine thousands of simulated microbial communities, showing that similarity-based and naïve diversity profiles only agreed approximately 50% of the time in their classification of which sample was most diverse. This is a strong argument for incorporating similarity information and calculating diversity with a range of emphases on rare and abundant species when quantifying microbial community diversity.

**Conclusions:**

For many datasets, diversity profiles provided a different view of microbial community diversity compared to analyses that did not take into account taxa similarity information, effective diversity, or multiple diversity metrics. These findings are a valuable contribution to data analysis methodology in microbial ecology.

## Background

With the widespread use of culture-independent, high-throughput sequencing technologies, ecologists have begun to describe the diversity of microbial communities that were previously difficult to detect e.g., [1-3]. Given the newness of these data types and the fact that the aims and goals of microbial studies are usually similar to those of macro-ecology, microbial ecologists often use methods from classical community ecology to analyze their data. These include Shannon’s H [4], Berger-Parker Evenness [5], rarefaction, and ordination [6].

While the use of established ecological metrics to analyze microbial diversity may sometimes be appropriate [7], the data produced by ecologists surveying macro-organismal communities differ from data obtained by high-throughput sequencing of microbial communities in three key ways. First, in contrast to plant and animal assemblages, microbial assemblages are typically made up of more than one domain of life, thus necessitating the ability to quantify diversity across very disparate organism types. Second, many classical indices assume ecological communities are composed of unique species. However, traditional biological species concepts do not fit the natural histories of many microbial taxa that routinely undergo non-homologous recombination [8-10] and sometimes lack sexual reproduction. (It is worth noting that the concept of species is widely questioned for macro-organisms as well [11].) Finally, unlike with macro-organisms, researchers are often unable to directly observe and characterize microbes and their traits *in situ*[12,13]. The taxonomic/phylogenetic and functional genes of environmental microbes are now commonly sequenced, but it is still very difficult to link the taxonomy of an individual microbe to the environmental functions it carries out.

These differences create methodological issues when discrete, taxonomic-based metrics are used to analyze microbial community datasets. The culture-independent approaches employed by microbial ecologists usually survey a variety of genes, intergenic spacers, and transcripts, which are typically classified into discrete, taxonomic bins called Operational Taxonomic Units (OTUs). Homologous genetic fragments that share less than a certain percentage of nucleotide polymorphisms are classified as being in the same genus or species (e.g., 97% similarity of the 16S gene is widely uses for “species”) [14-16]. This cutoff fails to adequately include the homology (and thus shared ecological function) with which the species concept was originally conceived.

The limitations of applying traditional diversity indices to microbial datasets lacking clear species delineations leave a number of questions: How can we quantify diversity using methods that are better suited for microbial datasets which span multiple domains of life? Does including similarity in our analyses change our interpretation of patterns of microbial diversity? What is the utility of including multiple dimensions of microbial diversity (i.e., taxonomic and phylogenetic) in our analyses?

One promising new way to analyze microbial community diversity and address these questions is through the use of diversity profiles, which were recently developed by Leinster & Cobbold [17,18]. These profiles are graphs that are used to display effective numbers of diversity (i.e., effective diversities). Effective diversities are mathematical generalizations of previous indices that behave much more intuitively, satisfying a number of desirable mathematical properties that provide meaningful percentage and ratio comparisons [19]. This is useful because many indices that have been traditionally used to describe macro-organismal community diversity and evenness can be quantitatively unintuitive (Inverse Simpson’s Diversity Index, Shannon’s Entropy, Gini-Simpson Index, etc.). For example, a community comprised of 10 hawks and 10 hummingbirds might experience a 50% decrease of both species, resulting in five hawks and five hummingbirds, but this change would not manifest as a 50% decrease in either Simpson Diversity or Shannon Diversity. Due to this, Hill [19] and later Jost [20] formulated effective number diversity metrics, which are simple entropies weighted by an order parameter, q. As the *q* parameter increases, the relative weight given to rare taxa in diversity index calculations declines. The effective diversity of order zero (*q* = 0) is equivalent to species richness (the total number of entities), order 1 is proportional to the Shannon index, and *q* = ∞ is a measure of pure evenness [17].

Diversity profiles significantly improve these previous calculations of effective diversity by adding community similarity information into diversity calculations, using a similarity matrix, **Z**. The term “similarity” is used by Leinster & Cobbold to refer to the degree of distance or difference between organisms. The similarity matrix can accommodate genetic similarity, phenotypic similarity, or any other biologically meaningful source of similarity between two or more entities. Incorporating this information into similarity-sensitive calculations of community diversity can greatly alter conclusions regarding diversity levels [17]. For example, when taking into account similarity between taxa, a bird community comprised of one hawk, one hummingbird, and one goose would be more diverse than a community of three distinct hummingbird species. However, if similarity between taxa were not taken into account, these communities would be classified as equally diverse.

For microbial communities, which are often characterized by phylogenetic molecular markers, the use of a metric based on the average evolutionary relatedness of a community conveys more information on the uniqueness and potential function of that community than does a discrete, OTU-based approach [21]. Recent work by Chao and colleagues [18], which expands on research by Faith [22], develops a measure of effective phylogenetic diversity. Effective phylogenetic diversity scales traditional diversity metrics by the hypothesized shared evolutionary history between taxa. Calculating phylogenetic diversity requires scaling raw taxonomic diversity by the shared evolutionary branches in a phylogeny. These branches can be either time-calibrated (ultrametric) or non-ultrametric. Even if a phylogeny is unavailable, the inclusion of cladistic data can be meaningful, if they accurately model shared ancestry within the study community. If the relative abundances of taxa or sequences are known, branches can also be weighted by abundance to compare the phylogenetic evenness among samples [23].

Given the differences between microbial and macro-organismal community data, the primary objective of this study was to evaluate the use of diversity profiles when analyzing microbial assemblages to determine whether the inclusion of similarity data (in our case, phylogenetic data) changes our interpretation of experimental and observational data. First, to explore whether diversity profiles alter our interpretation of microbial diversity data, we calculated diversity profiles for four datasets from different environments containing all domains of life and viruses. For comparison purposes, four statistics of pairwise community dissimilarity were calculated for the microbial datasets and plotted as dendrograms. Because diversity profiles can take into account the similarity of taxa and the relative importance of rare versus abundant taxa, we sought to evaluate how incorporating the phylogenetic similarity of taxa provides a different view of microbial diversity compared to traditional taxonomy-based metrics.

Second, we looked for evidence of bias and robustness of phylogenetic diversity profiles using simulated communities. We created numerous communities that varied in their rank abundance distributions, tree topologies, and whether ultrametric or non-ultrametric trees were used. Tree topologies were also simulated to create communities that spanned a large range of tree balances. Tree balance is determined by evolutionary processes, in particular lineage divergence and extinction rates and patterns, which differ greatly among real microbial communities [24]. We wanted to compare how “naïve” diversity profiles (what Leinster & Cobbold term calculations that do not take taxa similarity information into account [17]) and similarity-based diversity profiles are influenced by the topological characteristics (e.g., tree ultrametricity, tree balance) of the sampled communities. We tested the concordance between taxonomic and phylogenetic measures of diversity and composition. We predicted that since OTU-based metrics are discrete transformations of phylogenetic measures, they would generally agree. Simulations (and real data) were also used to test whether this concordance is correlated with aspects of the sampled community including aspects of its phylogenetic topology, richness, and abundance distribution. Our analyses indicate that phylogenetic diversity profiles provide insights into microbial community diversity that would not be discernible with the use of traditional univariate diversity metrics.

## Methods

### Diversity profiles

Diversity profiles were calculated for experimental, observational, and simulated microbial communities, as presented in detail by Leinster & Cobbold [17]. Briefly, consider a fully sampled community that contains *S* unique species. The relative abundances of the species are calculated by *p*_1_, . . . , *p*_*s*_, such that *p*_*i*_ ≥ 0 and $\sum_{i=1}^{S}\mathrm{pi}=1$. Because *p*_*i*_ ≠ 0, diversity profiles consider only species that are actually present in a community.

Information regarding the similarities between species in the community is taken into account by a matrix **Z** = (*Z*_*ij*_). The matrix has dimensions *S* X *S*, and *Z*_*ij*_ measures the similarity between the *i*th and the *j*th species. Similarity is scored such that 0 ≤ *Z*_*ij*_ ≤ 1, so that 0 represents complete dissimilarity between two species and 1 represents identical species. When similarity information is not available, or authors do not wish to include it, *Z*_*ij*_ = 1 in all cases, and this results in a naïve calculation.

Diversity profiles were then calculated across the range of a sensitivity parameter, *q*, for the values of 0 ≤ *q* ≤ ∞. At low values of *q*, such as *q* = 0, calculations of diversity are sensitive to rare taxa, and as *q* moves toward ∞, diversity calculations become more and more insensitive to the contributions of rare taxa.

For *q* ≠ 1, ∞, the diversity profile calculation is thus ${}^{q}D^{Z}\left(p\right)={\left(\sum \mathrm{pi}{\left(Zp\right)}_{i}^{q-1}\right)}^{\frac{1}{1-q}}$ where ${\left(Zp\right)}_{i}=\sum_{j=1}^{S}Z_{\mathrm{ij}}p_{j}$. The resulting ^*q*^*D*^**Z**^(**p**) is an effective number, and for certain values of *q* and **Z**, ^*q*^*D*^**Z**^(**p**) corresponds to a commonly used diversity index. For example, for naïve diversity profiles that do not take into account similarity between species, *q* = 0 is equivalent species richness, *q* = 1 is proportional to Shannon Diversity [4], *q* = 2 is proportional to 1/*D* (inverse Simpson Diversity) [25], and as *q* moves toward ∞, it is a measure of 1/Berger-Parker Evenness [5].

We calculated diversity profiles for 0 ≤ *q* ≤ 5. When plotting the profiles, we created larger insets for 1 ≤ *q* ≤ 2 [26]. For a more detailed description of the formulae used to calculate diversity profiles (e.g., their relationship to well-known diversity metrics, their potential benefits in diversity studies, examples of diversity profiles applied to macro-organism community datasets), refer to Leinster & Cobbold’s work [17].

### Environmental microbial datasets

Diversity profiles were used to quantify the diversity of four microbial datasets obtained from different environments containing bacterial, archaeal, fungal, and viral communities. The original four studies were conceived independently by co-authors of the current study, and we utilized these existing datasets to explore applications of diversity profiles to microbial community data. Providing complete details of each study is beyond the scope of the current study, but we have included brief descriptions of the studies’ methods below, and the research questions and hypotheses that shaped the design of each study are detailed in Table 1. We have also provided predicted outcomes of each of the studies, based on data and hypotheses from the original studies (Table 2). For further details of each study, please refer to the publications cited below.

**Table 1:** Research questions and hypotheses that shaped the design of the four environmental microbial community datasets

	***Research questions***	***Hypotheses***
*Acid mine drainage bacteria and archaea*	1) Are environmental (Env) samples more diverse than bioreactor (BR) biofilms?	H1: Bioreactor growth conditions usually have a higher pH than the environment, and the geochemistry of the drainage might differ from growth media. Thus, environmental biofilms are expected to be more diverse than bioreactor-grown biofilms.
	2) Is biofilm diversity higher at higher stages of biofilm development?	H2: As biofilms begin to establish, early growth-stage biofilms are expected to be less diverse. As they mature, more organisms join the community, increasing diversity.
*Hypersaline lake viruses*	1) How do viral diversities change across spatiotemporal replicates?	H1: Viral diversity will be greatest in pools with larger volume (2010A and 2007A samples).
		H2: Community dissimilarity will cluster by site, then by year.
*Subsurface bacteria*	1) Does acetate addition affect the diversity and composition of soil microbial communities?	H1: Acetate addition will stimulate growth of a subset of the microbial community capable of using it as an electron donor.
	2) Does vanadium addition affect the diversity and composition of soil microbial communities?	H2: Vanadium addition will reduce the diversity and evenness of the communities and favor those who can both use acetate as an electron donor and vanadium as an electron receptor and/or tolerate vanadium at high concentrations.
*Substrate-associated soil fungi*	1) How do plant community type (forest vs. grassland), substrate type (wood vs. straw), and time (6 months vs. 18 months) affect saprotrophic fungal assemblages?	H1: Wood substrates will be more diverse than straw substrates, because the wood substrate is more complex and requires a larger group of fungi to decompose it compared with a simpler substrate, such as straw.
		H2: Plant community type will have a greater effect on diversity than substrate type or time, because it will determine which fungi can colonize a substrate.

**Table 2:** Results of the diversity profiles for the four environmental microbial community datasets

	***Treatment***	***Naïve profiles results***	***Was this predicted?***	***Similarity profiles results***	***Was this predicted?***
*Acid mine drainage bacteria and archaea*	HiSeq	BR less diverse than most Env. samples	Yes	BR less diverse than Env. samples	Yes
		High GS only more diverse than early GS for Env-1	No	Highest GS (GS 2) is most diverse of all samples	Yes
	GAIIx	BR more diverse than Env-2, but less than Env-4	No	Env. samples mostly more diverse than BR	Yes
		Higher GS is less diverse than lower GS for BR	No	Highest GS is most diverse of all samples	Yes
*Hypersaline lake viruses*	N/A	Diversity greater in larger pools	Yes (2010A for 2/3 genes; not true for Cluster 667)	Diversity greater in combined 2007A samples and/or 2010A	Yes
*Subsurface bacteria*	N/A	Background > Acetate > Vanadium + acetate	Yes	Background ≈ Vanadium + acetate > Acetate	No
*Substrate-associated soil fungi*	Grassland	At all *q*: Wood T2 > Wood T1 > Straw T1 > Straw T2; No crossing along *q*	Yes	Straw T2 least diverse at all *q*	Yes
				At *q* = 0, Straw T1 has second lowest diversity, but by *q* = 3, has highest diversity	No
				Wood T2 > Wood T1 at all *q*	Yes
	Forest	At all *q*: Wood T1 > Straw T1 > Wood T2 > Straw T2; No crossing along *q*	No	At all *q*: Straw T1 > Wood T1 > Wood T2 > Straw T2; No crossing along *q*	No

#### Acid mine drainage bacteria and archaea

Total RNA was purified from eight environmental biofilm communities, collected from the Richmond Mine at Iron Mountain, Northern California in 2010 and 2011. In addition, total RNA was extracted from five biofilms grown in laboratory bioreactors using Richmond Mine inoculum in 2009 and 2010. Biofilms were collected or harvested at varying stages of development, ranging from early (GS0), mid (GS1), and late (GS2), as described previously [27].

RNA from all 13 samples was converted to cDNA and subject to Illumina library preparation and sequencing at the University of California Davis. Six environmental samples (from locations Env-1, Env-2, Env-3) and two bioreactor samples were sequenced using the HiSeq 2500 Illumina platform. Two environmental samples (from locations Env-2 and Env-4) and three bioreactor samples were sequenced using the GAIIx Illumina platform. A total of 256 million 75–100 bp long-reads were mapped to the small subunit (SSU) rRNA Silva database (including Archaea, Bacteria and Eukarya) with a similarity cutoff of 97% identity. SSU rRNA reads were then assembled using Cufflinks [28], and clustered at 97% identity using uclust [29]. SSU gene sequences were aligned using the SINA aligner webserver, and a phylogenetic tree was constructed using FastTree with options -gtr -nt -gamma. Normalized counts values obtained from Cufflinks were used as a measure of abundance of SSU rRNA genes sequences, as described earlier [27].

#### Hypersaline lake viruses

As previously described in detail [30,31], eight surface water samples were collected from two locations (A and B) within hypersaline Lake Tyrrell, Victoria, Australia (~330 g/L NaCl), with dates, locations, time scales, and sample IDs as follows: January 2007 (two samples, site A, two days apart, 2007At1, 2007At2), January 2009 (one sample, site B, 2009B), January 2010 (one sample, site A, 2010A; four samples, site B, each approximately one day apart, 2010Bt1, 2010Bt2, 2010Bt3, 2010Bt4). In the summer, when samples were collected, the lake dries and leaves residual briny “pools” in a few isolated sites. Sites A and B are different pools ~300 m apart.

Post-0.1 μm filtrates were concentrated via tangential flow filtration for the collection of viral particles, followed by DNA extraction and metagenomic sequencing. 454-Titanium technology (~400 bp reads) was used to sequence samples 2010Bt1 and 2010Bt3, and Illumina GAIIx paired-end technology (~100 bp reads) was used to sequence the remaining six samples, for a total of 6.4 billion bp. Previous analyses of these data show that there was no observable difference between the 454-Titanium data and the Illumina data [30-32]. Each sample was assembled separately via Newbler [33], ABySS [34], or Velvet [35]. Genes from all contigs >500 bp were predicted with Prodigal [36], and predicted genes longer than 300 bp were retained and clustered at 95% nucleotide identity, using uclust [30]. Corresponding predicted proteins were separately 1) annotated with InterProScan [37] and 2) clustered at 40% amino acid identity, using uclust [30]. In the absence of a universal marker gene, six viral “OTU groups” were chosen [32]. Three were used for this study: methyltransferases (the most abundant annotation), concanavalin A-like glucanases/lectins (the most abundant annotation likely to be exclusive to viruses), and Cluster 667 (one of the largest protein clusters of unknown function). Proteins for each OTU group were aligned with MUSCLE [38], and a phylogenetic tree was constructed from the alignments, using FastTree [39] with default parameters.

#### Subsurface bacteria

DNA was extracted from five sediment samples taken from *in situ* flow-through columns buried in sampling wells in a shallow, uranium and vanadium-contaminated aquifer in Rifle, Colorado as described previously [40]. Samples were from background sediment (B), sediment stimulated with carbon and vanadium addition (V1, V2), and sediment stimulated with carbon addition alone (A1, A2). Universal primers and gradient PCR were used to amplify the 16S small subunit ribosomal RNA gene from the organisms sampled.

HiSeq Illumina paired-end technology was used to sequence 2.7 megabases of PCR product at the University of California, Davis. The sequencing consisted of 26,954,412 100-base pair reads. Reads were mapped to reference sequences from the Silva database with the EMIRGE iterative algorithm [41,42]. The genes were aligned to each other, using the SSU-align software [43]. The alignment was automatically masked with the ssu-mask program. Bacterial OTUs were then clustered at a 97% nucleotide identity cutoff, using usearch [29]. A phylogenetic tree was constructed with the aligned sequences via the FastTree maximum likelihood method with options –gtr –nt and 1000 iterations of the FastTree bootstrap [40,44].

#### Substrate-associated soil fungi

The goal of this study was to determine if substrate, space, time or plant community were the major determinants of fungal saprotrophic community composition. Sampling of buried substrates (straw and wood blocks) occurred on Bolinas Ridge on Mount Tamalpais in Marin County, California, USA along four 10 × 10 m blocks in 2007 and 2008, as previously described [45]. Two blocks were in the coastal grassland and two blocks were in the adjacent forest dominated by *Pseudotsuga menziesii*. The region is characterized as having a Mediterranean climate with a seasonal summer drought. DNA was extracted from 32 bait bags filled with sterile wheat straw and 32 small conifer wood blocks that had been buried (<10 cm) in both the grassland and forest blocks (16 straw samples and 16 wood samples were buried in each plant community type). Half of the straw and wood substrates were buried for six months (time point 1), while the others were buried for 18 months (time point 2).

DNA was purified, and the LSU region (LROR_F [46]/LR5-F [47]) was PCR amplified with 10 bp MID barcodes. 454 Pyrosequencing 1/8 of a plate resulted in a total of 123,117 LSU sequences. Reads were trimmed and filtered using the QIIME software [48]. Non-fungal taxa, sequences that resulted in no BLAST matches, and singletons were removed from the analysis. OTUs were conservatively determined at 95% sequence similarity. FastTree [39] was used for phylogenetic tree building in QIIME. For community analyses, only samples with at least 600 LSU sequence reads were included.

### Analysis of datasets

Diversity profiles for each dataset were calculated using an R code adapted from Leinster & Cobbold [17]. For each community, both naïve diversity profiles and diversity profiles that took into account similarity information derived from the community phylogenies were calculated. The resulting profiles were then compared and analyzed. Specifically, we sought to identify differences between naïve and phylogenetic measures of diversity and community composition that would affect our interpretation of patterns in the data. The topology of the phylogenetic trees constructed from these datasets were quantified using Colless’ I tree balance statistic [49] with Yule normalization; high values of Colless’ I correspond to imbalanced, asymmetric trees and low values correspond to more balanced trees (Table 3).

**Table 3:** Yule normalized Colless’ I tree balance calculations for the four environmental microbial community datasets

	**Number of tips**	**Yule normalized colless’ I**
Acid mine drainage bacteria and archaea	158	5.27
Hypersaline lake viruses: Cluster 667	71	0.33
Subsurface bacteria	10405	34.85
Substrate-associated soil fungi	1973	9.81

In order to compare the diversity calculations produced by diversity profiles to more traditional calculations of community composition for the same datasets, four different statistics of pairwise community dissimilarity were computed (abundance-weighted Jaccard, unweighted Jaccard, abundance-weighted UniFrac, and unweighted UniFrac). The Jaccard index, is the ratio of the number of taxa shared between two samples to the total number of taxa in each sample and then this ratio subtracted from one [50]. Pairwise phylogenetic dissimilarity for each sample was calculated using the UniFrac method [51]. This metric measures the proportion of unshared phylogenetic branch lengths between two samples. Ward’s minimum-variance method [52] was used to complete hierarchical clustering on the samples based on each dissimilarity metric and plot them as dendrograms. Please see Additional file 1 for these results.

### Simulations

We simulated hundreds of microbial communities in order to better measure the degree to which differences between naïve and similarity-based diversity profiles are influenced by the abundance and phylogenetic distributions of microbial communities. Each simulated community was distributed according to one of four possible commonly fitted rank abundance distributions (Log Normal, Geometric, Log Series, or Uniform) and had a random phylogenetic tree topology. Tree topologies were simulated so as to create communities that spanned a large range of tree imbalances. Tree imbalance was quantified using Yule normalized Colless’ I tree balance statistic [49]. Lastly, all trees were simulated in both ultrametric and non-ultrametric versions to test the effects of branch lengths on the diversity profiles.

To look for systematic differences between naïve and phylogenetic diversity profiles, we repeatedly (100 times) took a random sample of OTUs from two simulated communities and calculated the proportion of times that the naïve and phylogenetic diversity profiles agreed on which random sample was more diverse. We analyzed whether agreement between naïve and similarity-based diversity profiles systematically differed based on numbers of OTUs sampled, whether trees were ultrametric or non-ultrametric, Fisher’s alpha diversity values, or tree imbalance values.

## Results and discussion

Given the potential limitations of applying traditional diversity indices to microbial datasets produced by high-throughput sequencing, we sought to evaluate microbial diversity using methods that might be better suited for microbial taxa that span multiple domains of life and multiple dimensions of diversity (e.g., taxonomic, phylogenetic). The advantages of using diversity profiles are that they encompass a number of other common diversity indices and allow for the incorporation of species similarity information.

We systematically tested diversity profiles as a metric for quantifying microbial diversity by analyzing four natural experimental and observational microbial datasets from varied environments that contained bacterial, archaeal, fungal, and viral communities. (Refer to Table 4 for summaries of these datasets.) For each of the four datasets, we specified plausible alternative hypotheses for the ecological drivers of each community’s diversity (Table 1), as well as expected results (Table 2, Additional file 1: Table S1). Additionally, we tested diversity profiles on the simulated microbial datasets.

**Table 4:** Summaries of the four environmental microbial community datasets

	***Dataset summary***	***Resulting data***
*Acid mine drainage bacteria and archaea*	Total RNA was collected from 8 environmental biofilms and 5 bioreactor biofilms at varying stages of development: early (GS0), mid (GS1), and late (GS2). RNA from all samples was converted to cDNA. 6 environmental and 2 bioreactor samples were sequenced using HiSeq 2500 Illumina. 2 environmental and 3 bioreactor samples were sequenced using GAIIx Illumina.	159 SSU-rRNA sequence fragments were identified in 13 biofilms. The number of reads and SSU-rRNA sequences assembled from the GAIIx and the HiSeq platforms differed greatly; thus the rarefied data from these sequencing methods were analyzed separately (HiSeq: Figure 2, GAIIx: Additional file 1: Figure S1).
*Hypersaline lake viruses*	8 surface water samples were collected within a hypersaline lake as follows: Jan. 2007 (2 samples, site A, 2 days apart, 2007At1, 2007At2), Jan. 2009 (1 sample, site B, 2009B), Jan. 2010 (1 sample, site A, 2010A; 4 samples, site B, each ~1 day apart, 2010Bt1, 2010Bt2, 2010Bt3, 2010Bt4). 454-Titanium was used to sequence samples 2010Bt1 and 2010Bt3. Illumina GAIIx was used to sequence the remaining 6 samples.	630 methyltransferase genes, 411 concanavalin A-like glucanases/lectins, and 71 putative genes falling under Cluster 667 were assembled from the viral metagenomic reads (Methyltransferase: Additional file 1: Figure S2, Concanavalin: Additional file 1: Figure S3, Cluster 667: Figure 1).
*Subsurface bacteria*	DNA was extracted from 5 sediment samples taken from *in situ* flow-through columns buried in sampling wells in a shallow, uranium and vanadium-contaminated aquifer: background sediment (B), sediment stimulated with carbon and vanadium addition (V1, V2), and sediment stimulated with carbon addition alone (A1, A2). HiSeq Illumina was used to sequence 16S SSU-rRNA PCR product.	25,966 OTUs were identified from 5 subsurface samples (Figure 3).
*Substrate-associated soil fungi*	DNA was extracted from 32 straw bait bags and 32 wood blocks that were buried in grassland and forest (16 straw and 16 wood in each). Half of the substrates were buried for six months (time point 1) and half for 18 months (time point 2). 454-Titanium was used to sequence the PCR amplified LSU region.	508 total OTUs were identified within all substrate samples (Grassland: Figure 4, Forest: Additional file 1: Figure S4).

### Naïve microbial diversity comparisons may vary with the sensitivity parameter, q

Diversity profiles calculated from the experimental and observational datasets provided insights into microbial community diversity that would not be perceivable through the use of a classical univariate diversity metric. The sensitivity of diversity profiles to rarity greatly affected diversity measurements. Richness calculations count all taxa equally, greatly overestimating the contribution of rare taxa to diversity, whereas diversity measurements at high values of *q* are insensitive to the contribution of rare OTUs. Diversity profiles illustrate this stark contrast and highlight the question of the importance of ultra-rare taxa, the “rare biosphere” of Sogin et al. [53]. Previously, these ultra-rare taxa were not included in diversity calculations because they were not detected using older methods of measuring microbial taxa (clone libraries, low depth sequencing, DGGE, etc.). Newer techniques such as deep short-read sequencing have revealed the existence of these taxa, but introduced more bias into older diversity indices such as species richness calculations. The datasets analyzed here demonstrate the importance of rare taxa.

**Figure 1:**
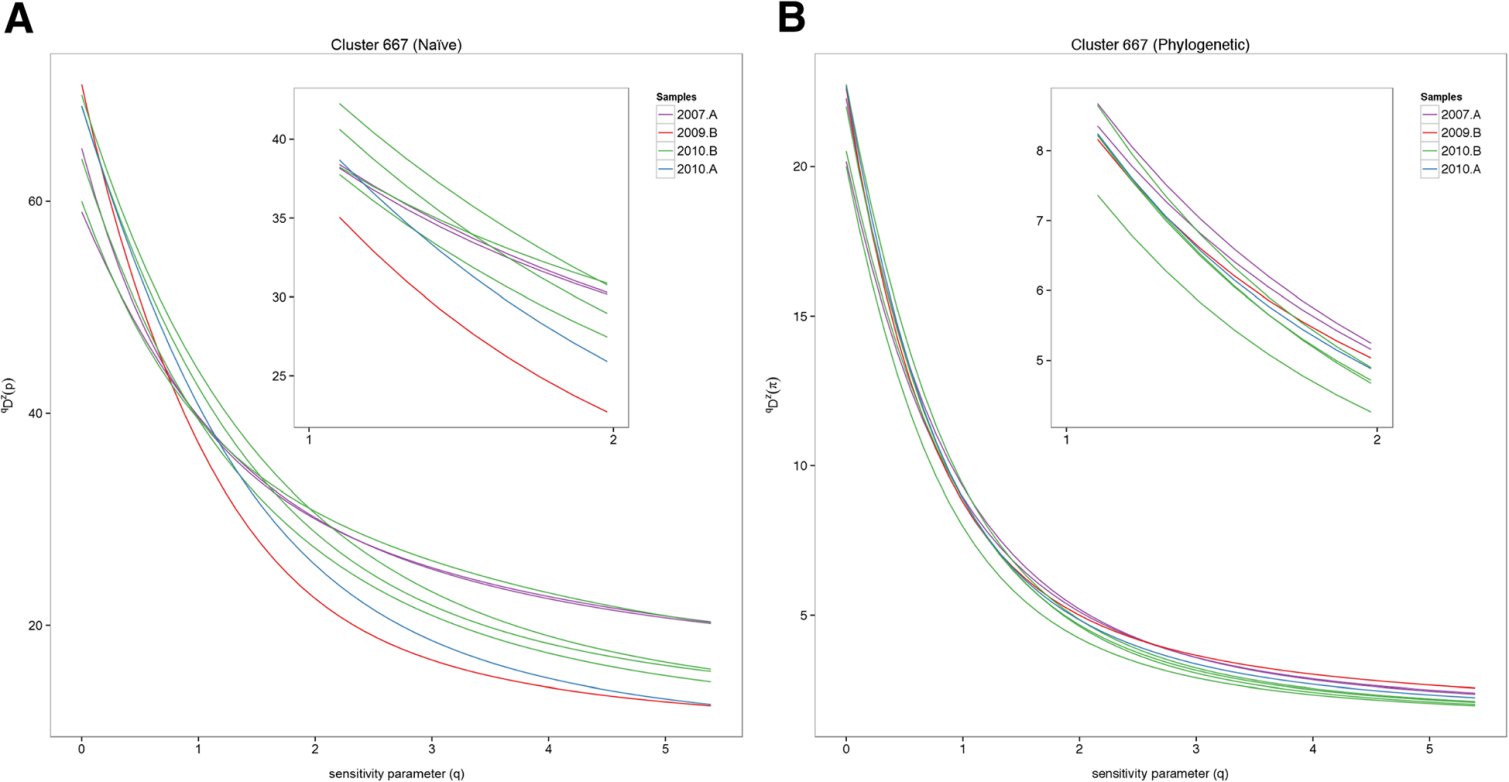
**Hypersaline lake viruses Cluster 667 diversity profiles. (A)** Naïve and **(B)** similarity-based (phylogenetic relatedness) diversity profiles calculated for Cluster 667 from the hypersaline lake viruses data.

This is clearly indicated by the viral data from the hypersaline lake viruses dataset. For the viral gene clusters described in this study, there was some disagreement in the relative diversity rankings of samples across the range of *q* plotted in all three naïve diversity profiles (Table 1, Figure 1, Additional file 1: Figures S2, S3). First, if diversity of the putative genes falling under Cluster 667 were analyzed with the naïve analysis using only species richness (*q* = 0 in the diversity profile), the resulting calculations would have indicated that the 2009B sample was the most diverse (Figure 1). However, by *q* = 1 (which is proportional to calculating Shannon index) and for all higher values of *q*, the sample 2009B had the lowest diversity within the dataset. This change in ranking at higher values of *q* indicates that the 2009B sample had many rare taxa, because as *q* increases, the weight given to rare taxa in diversity profile calculations decreases [17]. Secondly, in the naïve diversity profile for the putative methyltransferase group, the lines representing the diversity of the 2007A, 2009B, and 2010B samples crossed each other numerous times between *q* = 0 and *q* = 5 (Additional file 1: Figure S2). Lastly, in the naïve profile for the putative concanavalin A-like glucanases/lectins group, the 2010B samples were as diverse as or more diverse than the 2007A samples at *q* = 0, but the diversity of 2010B samples dropped sharply and remained lower than all other samples after approximately *q* = 0.5 (Additional file 1: Figure S3). In the case of viral diversity, ultra-rare taxa play an important role in rapid evolution to allow new viruses to infect hosts that are constantly evolving defense mechanisms. Thus, diversity calculated at low values of *q*, which are sensitive to rare taxa, is the more appropriate measure of viral diversity.

**Figure 2:**
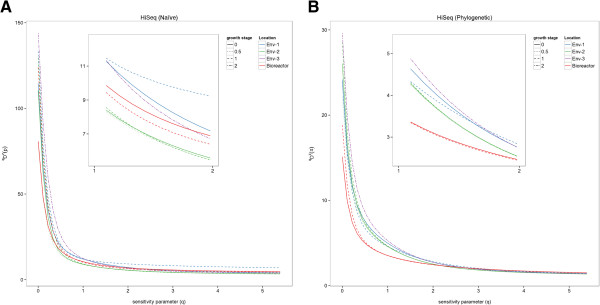
**Acid mine drainage bacteria and archaea** (**HiSeq**) **diversity profiles. (A)** Naïve and **(B)** similarity-based (phylogenetic relatedness) diversity profiles calculated from the acid mine drainage bacteria and archaea HiSeq data.

We see similar results for the acid mine drainage dataset. At *q* = 0 (species richness) in the naïve analysis, the Env-3 at growth stage 2 sample is the most diverse sample, but the sample’s diversity decreases and is surpassed by the growth stage 0 bioreactor sample and both Env-1 samples between *q* = 1 and *q* = 2 (Figure 2), demonstrating that the bioreactor and Env-1 samples were less even than the Env-3 sample at growth stage 2. Thus, for this dataset as well as for the hypersaline lake viruses dataset, evaluating the diversity of the microbial communities at multiple values of *q* leads to a different interpretation of the results and response to the original hypotheses (Table 1).

**Figure 3:**
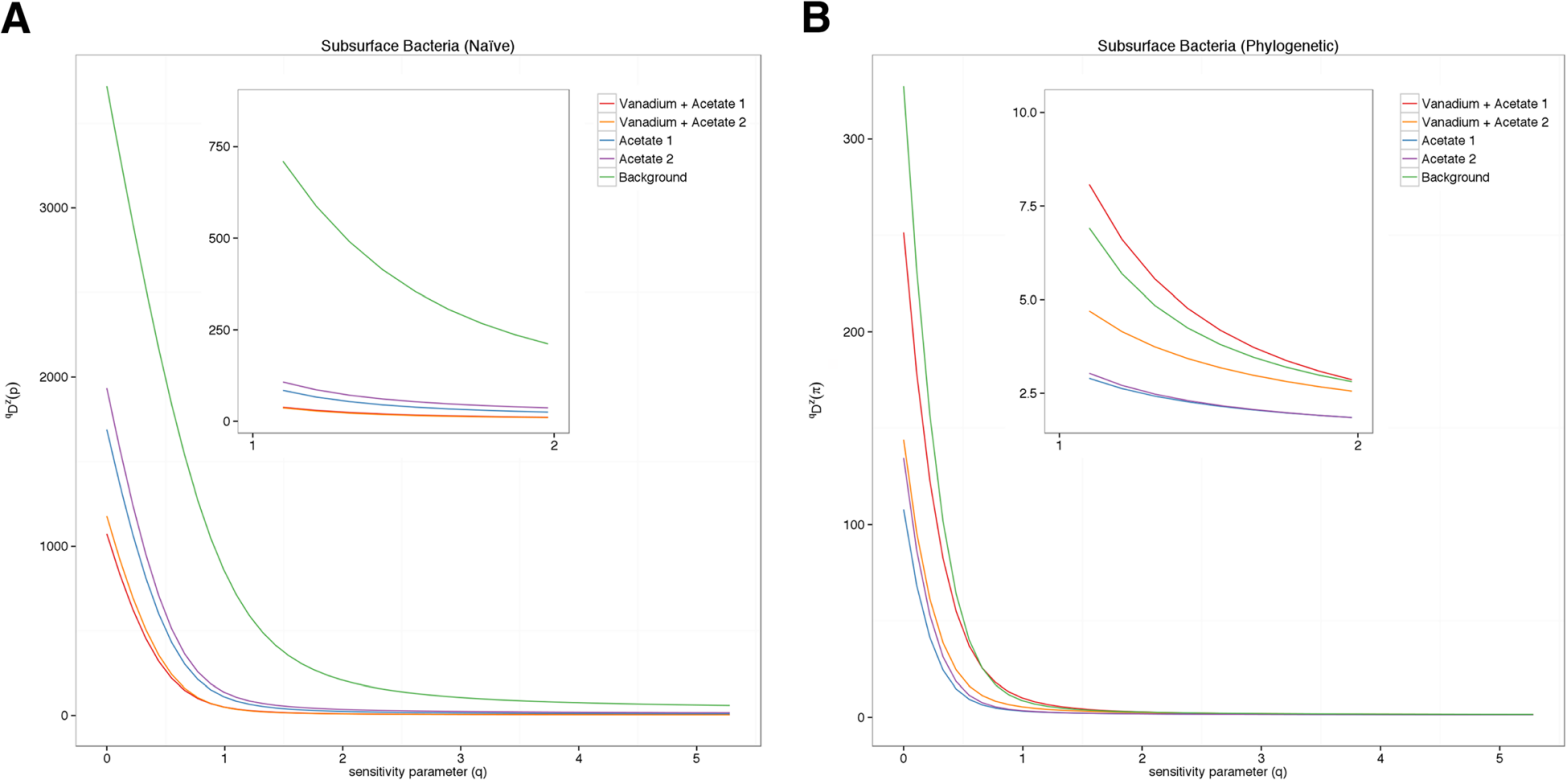
**Subsurface bacteria diversity profiles. (A)** Naïve and **(B)** similarity-based (phylogenetic relatedness) diversity profiles calculated from the subsurface bacteria data.

Diversity profiles do not always add new information to analyses of natural microbial datasets. In some cases, such as with the naïve profiles of the subsurface bacteria dataset, the most diverse samples in a dataset were always calculated as the most diverse, across the entire range of *q* in the naïve profile (Figure 3). Thus, whether we quantified diversity using species richness, Shannon diversity, or diversity profiles, we would arrive at the same result. In general, our findings provide evidence for the utility of diversity profiles to analyze microbial datasets, even when similarity information is not taken into account, because they allow researchers to visualize multiple diversity indices across the range of *q* in the same place after just one calculation. They also clearly provide information about the effects of rare species in a sample on diversity calculations.

### Similarity information may alter microbial diversity calculations

The analyses presented here demonstrate the value of using diversity profiles to incorporate phylogenetic diversity as a measure of taxa similarity into diversity calculations. For all four microbial datasets we analyzed, we saw key distinctions between naïve taxonomic diversity calculations and those that incorporated phylogenetic information. For example, in the subsurface bacterial dataset, naïve measurements of OTU richness for each treatment indicated that the background sample (no treatment) contained the highest diversity for all values of *q* (Table 2, Figure 3A). Additionally, naïve measurements of both acetate-only samples were more diverse than the samples amended with both acetate and vanadium. These were the expected results as the experiment involved a treatment that should have selected for taxa that could use acetate as a carbon source and vanadium as an energy source (Table 1).

Phylogenetic results, on the other hand, suggested that the vanadium-acetate samples were as diverse as background samples and more diverse than the acetate-only treatments (Table 2, Figure 3B), indicating that perhaps the ability to use vanadium for energy or to tolerate its presence was more phylogenetically widespread than expected. Previous analysis of these data using Faith’s phylogenetic diversity metric found the background sediment to be most phylogenetically diverse [40], which Figure 3B also shows at *q* = 0. However, the crossing of the background sample and the acetate and vanadium treated samples when 1 ≤ *q* ≤ 2 in Figure 3B indicates a greater diversity of common taxa in the treated sites. This indicates that adding abundance information to measures of phylogenetic diversity through the use of diversity profiles can add depth to the interpretation of diversity calculations.

In another example, in forest samples at T = 1 in the substrate-associated soil fungi dataset, wood substrates contained greater naïve taxonomic diversity. This higher diversity on wood substrates compared to straw substrates was hypothesized because the wood substrate is more complex and requires a larger group of fungi to decompose it compared with a simpler substrate, such as straw (Table 1). However, the wood substrates actually contained lower phylogenetic diversity than straw substrates (Additional file 1: Figure S4). These results indicate that the fungal communities growing on wood substrates contained more member taxa that were closely related to each other, because when phylogenetic similarity was included in diversity calculations, the diversity of wood substrate fungal communities decreased.

**Figure 4:**
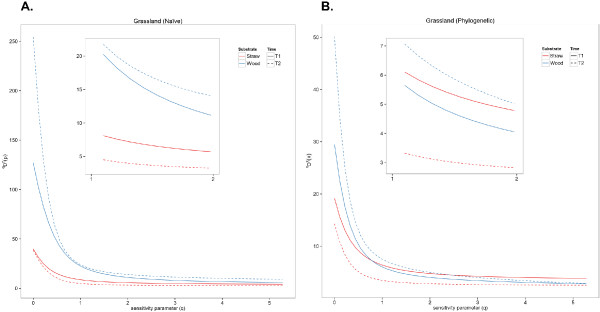
**Substrate-associated soil fungi grassland diversity profiles. (A)** Naïve and **(B)** similarity-based (phylogenetic relatedness) diversity profiles calculated from the substrate-associated soil fungi grassland data.

Similarly, when analyzing the grassland samples of the substrate-associated soil fungi dataset, the wood substrate samples contained greater naïve taxonomic diversity at both time points than the straw substrates (again, as hypothesized in Table 1), within the range of 0 ≤ *q* ≤ 5 (Figure 4A). However, when phylogenetic similarity was included, the fungi growing on straw substrates at T = 1 were more diverse than the fungi growing on wood substrates at T = 1, within the range of 1 ≤ *q* ≤ 5 (Figure 4B). This indicates that the fungal communities growing on straw substrates in the grassland at T = 1 contained taxa that were less closely related to each other (more phylogenetically diverse) than the taxa growing on wood substrates at T = 1, because when phylogenetic similarity was considered, the diversity of straw substrate fungal communities increased. There was also considerable overlap and crossing in the phylogenetic diversity profile between 1 ≤ *q* ≤ 3, which was not apparent in the taxonomic profile.

This demonstrated capacity of diversity profiles to incorporate effective phylogenetic diversity, as well as other measures of similarity between taxa, is particularly meaningful for analyzing microbial diversity data. Macro-organismal ecologists have long been concerned with the interactions between an organism’s traits and aspects of its ecology, such as its niche axes or its role in ecosystem processes [54-57]. Many macro-eukaryote traits, when mapped to phylogenies, show evidence for phylogenetic conservatism [58,59]. That is, certain traits are shared more often by closely related taxa than would be expected by chance. Even bacteria and archaea show evidence for trait conservatism, despite the role of non-homologous recombination in their evolutionary history [60,61]. This implies that the phylogenetic distribution of a microbial assemblage can, thus, influence ecosystem processes via differences in the suite of traits present. Phylogenetic trait conservatism in microbes also has practical implications, such as potentially guiding current research in drug discovery or biodegradation [62-64].

Diversity analyses of environmental microbial samples can span all domains of life. It is thus highly desirable to evaluate and critically assess a method that can address the diversity of a microbial assemblages effectively across domains, as well as across samples with substantial differences in rare membership, while using a full complement of the information contained in DNA and RNA sequence analysis. As there is no universal marker gene for viruses, there are no robust means of determining viral phylogeny from community sequencing data. Apart from a few groups of well-characterized viruses, it is difficult to characterize viral phylogenetic relationships at all. In our similarity-based profiles, we assume that sequence and, therefore, tree similarity are proxies for phylogenetic similarity. This is reasonable for phylogenetically informative genes, such as the SSU rRNA genes in cellular organisms. However, in the case of genes from the hypersaline virus dataset, and any other viral metagenomic data to which diversity profiles may be applied, this is almost certainly not true. In our application of sequence similarity-based diversity profiles to viruses, we essentially (incorrectly) inferred phylogeny from functional genes that are likely subject to extensive horizontal gene transfer. While these genes are still informative in that they might correspond to the host range and thus the viruses’ community function, we suggest that naïve diversity profiles will be more useful for analyses of viral assemblages than similarity-based profiles, unless a more robust means of determining viral phylogeny is discovered.

### Diversity profile simulations

The four microbial datasets analyzed in this study were well-suited to test the application of diversity profiles to microbial data, particularly because they spanned multiple domains of life and dimensions of diversity. However, while treatment replicates were included in the diversity profiles for two of the datasets (hypersaline lake viruses, subsurface bacteria dataset), they were not included for the other two datasets. Therefore, statistical tests were not performed to determine whether the diversity of a group of samples was significantly higher or lower than other groups. Additionally, while it is noteworthy that we analyzed four unique microbial datasets within this study, our conclusions of how diversity profiles perform when analyzing microbial data were limited based on this relatively small number of datasets.

In order to address these shortcomings of the data, we simulated microbial communities. Simulations allowed us to utilize diversity profiles at the scale of hundreds of simulated microbial datasets with a range of abundance distributions and phylogenetic tree topologies, so that analyses were carried out with greatly increased replication. The major finding from this simulation study is that when we repeatedly took a random sample of OTUs from two simulated communities and compared their diversity, naïve and similarity-based diversity profiles agreed only approximately 50% of the time in their classification of which sample was most diverse (95% confidence interval was 29.8% to 74.6%, mean was 52.2% across all experiments). This finding is a strong argument for analyzing more than taxonomic diversity when quantifying the diversity of microbial communities. The evolutionary or phylogenetic distance among members of microbial consortia is arguably foundational in assessing diversity of these nodes of life that span the domains. It appears that microbial diversity analyses should include similarity information whenever it is available or its omission should be appropriately justified. Such similarity information need not include continuous evolutionary distances, but could be as simple as assigning similarity values based on general taxonomic group.

**Figure 5:**
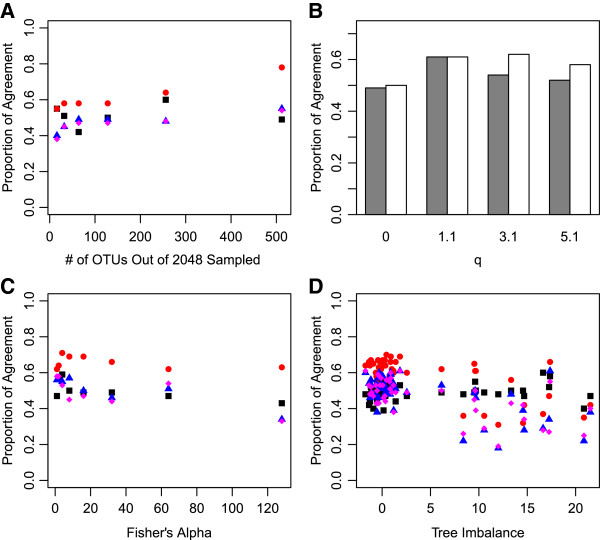
**Agreement between naïve and similarity-based diversity profiles for different simulated communities. (A)** For different numbers of OTUs sampled from the total pool of 2048, **(B)** for ultrametric (grey) and non-ultrametric trees (white), **(C)** for communities with different Fisher’s alpha diversity values, **(D)** for communities with different tree imbalances. For panels **(B)**, **(C)**, &**(D)** sampled communities sized was 256; **(A)**, **(B)**, &**(C)** tree imbalance was 9.54; **(A)**, **(B)**, &**(D)** community abundance distribution was logseries with a Fisher’s Alpha of 1. Proportion of agreement is based on 100 simulations. “black square symbol” (*q* = 0), “red circle symbol” (*q* = 1.1) “blue triangle symbol” (*q* = 3.1), “magenta triangle symbol” (*q* = 5.1).

Our simulations showed that, to some extent, the choice of *q* did effect the agreement between naïve and similarity-based diversity calculations. Generally speaking, for small positive *q* values it appears that there was greater agreement between naïve and similarity-based diversity calculations. These differences were statistically significant when the difference in proportion of agreement between two *q* was ~ 0.15 (based on Z test for two population proportions). Turning to the impacts of tree typology and sample relative abundance distributions, our results showed that the percent agreement between the naïve and similarity-based diversity calculations decreased slightly with increasing skewed abundance distributions (Figure 5C) and increasing tree imbalance (Figure 5D). This finding is significant because, while tree shape changes greatly between different sized trees [65], skewed abundance distributions [66,67] and higher tree imbalances [25,65] are likely better representations of the majority of true environmental communities than perfectly balanced abundance distributions and phylogenies would be. In contrast, the percent of agreement increased slightly with increasing sample size (Figure 5A) and the use of non-ultrametric trees (Figure 5B), which are also likely good representations of the majority of true environmental microbial communities that may include thousands of OTUs e.g., [68] and may produce undated non-ultrametric trees. Since these simulations of phylogenetic trees with characteristics that resemble those of real datasets showed both slight increases and decreases in the percent agreement between the naïve and similarity-based diversity calculations, the percent agreement between naïve and similarity-based diversity calculations for real datasets is probably approximately 50%.

## Conclusions

This study explored whether similarity-based diversity profiles can aid our interpretation of microbial diversity. The findings indicate that the use of phylogenetic metrics and effective numbers can provide additional insight into the diversity of microbial communities when combined with naïve analyses that do not take into account similarity information or multiple diversity metrics. The ongoing question of how to best analyze microbial community datasets is paramount to deducing the processes that affect the composition and function of microbial communities. The type of information and metric used to measure biological diversity in any study of microbial diversity is a decision that must be well-justified prior to hypothesis testing instead of being made arbitrarily based solely on which metrics are popularly used by plant and animal ecologists. This justification, in turn, should be based on evidence produced by work, such as this study, that has systematically tested the efficacy and utility of these diversity metrics under a range of situations.

## Availability of supporting data

The R code adapted from Leinster & Cobbold [17] and used to calculated diversity profiles is available for download and use at https://gist.github.com/darmitage. The hypersaline lake viruses raw sequencing reads are available in the NCBI BioProject (accession number PRJNA81851, http://www.ncbi.nlm.nih.gov/bioproject/?term=PRJNA81851). The subsurface bacteria dataset is available at: http://banfieldlab.berkeley.edu/SOM/yelton2012/.

## Abbreviations

OTUs: Operational taxonomic units; SSU: Small subunit.

## Competing interests

The authors declare that they have no competing interests.

## Authors’ contributions

HMD, DWA, RAD, JBE, DSAG, APY, MKF, and MDP conceived of the study. RAD, MKF, and MDP led the study’s design and coordination. JBE, DSAG, AY, and JK designed the experiments and collected the data for the four environmental microbial datasets. DWA and MDP designed the simulations, and MDP carried out the simulations. All authors analyzed the results. HMD, DWA, and MDP drafted the manuscript. All authors read and approved the final manuscript.

## Supplementary Material

Additional file 1: Table S1 – Results of the community composition analyses (Jaccard and Unifrac) for the four environmental microbial community datasets. **Figure S1.** – Acid mine drainage bacteria and archaea (GAIIx) diversity profiles. **Figure S2.** – Hypersaline lake viruses methyltransferase diversity profiles. **Figure S3.** – Hypersaline lake viruses concanavalin A-like glucanases/lectins diversity profiles. **Figure S4.** – Substrate-associated soil fungi forest diversity profiles. **Figure S5.** – Acid mine drainage bacteria and archaea (HiSeq) phylogenetic (UniFrac) and taxonomic (Jaccard) hierarchical dissimilarity clusters. **Figure S6.** – Acid mine drainage bacteria and archaea (GAIIx) phylogenetic (UniFrac) and taxonomic (Jaccard) hierarchical dissimilarity clusters. **Figure S7.** – Hypersaline lake viruses Cluster 667 phylogenetic (UniFrac) and taxonomic (Jaccard) hierarchical dissimilarity clusters. **Figure S8.** – Hypersaline lake viruses methyltransferase phylogenetic (UniFrac) and taxonomic (Jaccard) hierarchical dissimilarity clusters. **Figure S9.** – Hypersaline lake viruses concanavalin A-like glucanases/lectins phylogenetic (UniFrac) and taxonomic (Jaccard) hierarchical dissimilarity clusters. **Figure S10.** – Subsurface bacteria phylogenetic (UniFrac) and taxonomic (Jaccard) hierarchical dissimilarity clusters. **Figure S11.** – Substrate-associated soil fungi phylogenetic (UniFrac) and taxonomic (Jaccard) hierarchical dissimilarity clusters. Click here for file

## References

[B1] Roesch LFW Fulthorpe RR Riva A Casella G Hadwin AKM Kent AD Daroub SH Camargo FAO Farmerie WG Triplett EW Pyrosequencing enumerates and contrasts soil microbial diversity ISME J 2007 1 283 290 18043639 10.1038/ismej.2007.53 PMC2970868

[B2] Fulthorpe RR Roesch LFW Riva A Triplett EW Distantly sampled soils carry few species in common ISME J 2008 2 901 910 10.1038/ismej.2008.55 18528413

[B3] Fierer N McCain CM Meir P Zimmermann M Rapp JM Silman MR Knight R Microbes do not follow the elevational diversity patterns of plants and animals Ecology 2011 92 797 804 10.1890/10-1170.1 21661542

[B4] Shannon CE A mathematical theory of communication Bell System Technical Journal 1948 27 379 423 10.1002/j.1538-7305.1948.tb01338.x

[B5] Berger WH Parker FL Diversity of Planktonic Foraminifera in deep-sea sediments Science 1970 168 1345 1347 10.1126/science.168.3937.1345 17731043

[B6] Bent SJ Forney LJ The tragedy of the uncommon: understanding limitations in the analysis of microbial diversity ISME J 2008 2 689 695 10.1038/ismej.2008.44 18463690

[B7] Hill TCJ Walsh KA Harris JA Moffett BF Using ecological diversity measures with bacterial communities FEMS Microbiol Ecol 2003 43 1 11 10.1111/j.1574-6941.2003.tb01040.x 19719691

[B8] Taylor JW Jacobson DJ Kroken S Kasuga T Geiser DM Hibbett DS Fisher MC Phylogenetic species recognition and species concepts in fungi Fung Genet Biol 2000 31 21 32 10.1006/fgbi.2000.1228 11118132

[B9] Rosselló-Mora R Amann R The species concept for prokaryotes FEMS Microbiol Rev 2001 25 39 67 11152940 10.1111/j.1574-6976.2001.tb00571.x

[B10] Staley JT The bacterial species dilemma and the genomic-phylogenetic species concept Philos Trans R Soc Lond B Biol Sci 2006 361 1899 1909 10.1098/rstb.2006.1914 17062409 PMC1857736

[B11] Mishler BD Ayala FJ, Arp R Species are not uniquely real biological entities Contemporary Debates in Philosophy of Biology 2010 Oxford: Wiley-Blackwell 110 122

[B12] Tiedje JM Asuming-Brempong S Nüsslein K Marsh TL Flynn SJ Opening the black box of soil microbial diversity Appl Soil Ecol 1999 13 109 122 10.1016/S0929-1393(99)00026-8

[B13] Luo F Yang Y Zhong J Gao H Khan L Thompson DK Zhou J Constructing gene co-expression networks and predicting functions of unknown genes by random matrix theory BMC Bioinf 2007 8 299 10.1186/1471-2105-8-299 PMC2212665 17697349

[B14] Horner-Devine MC Lage M Hughes JB Bohannan BJM A taxa-area relationship for bacteria Nature 2004 432 750 753 10.1038/nature03073 15592412

[B15] O’Brien HE Parrent JL Jackson JA Moncalvo J-M Vilgalys R Fungal community analysis by large-scale sequencing of environmental samples Appl Environ Microbiol 2005 71 5544 5550 10.1128/AEM.71.9.5544-5550.2005 16151147 PMC1214672

[B16] Buée M Reich M Murat C Morin E Nilsson RH Uroz S Martin F 454 Pyrosequencing analyses of forest soils reveal an unexpectedly high fungal diversity New Phytol 2009 184 449 456 10.1111/j.1469-8137.2009.03003.x 19703112

[B17] Leinster T Cobbold CA Measuring diversity: the importance of species similarity Ecology 2012 93 477 489 10.1890/10-2402.1 22624203

[B18] Chao A Chiu C-H Jost L Phylogenetic diversity measures based on Hill numbers Philos Trans R Soc Lond B Biol Sci 2010 365 3599 3609 10.1098/rstb.2010.0272 20980309 PMC2982003

[B19] Hill MO Diversity and evenness: a unifying notation and its consequences Ecology 1973 54 427 432 10.2307/1934352

[B20] Jost L Entropy and diversity Oikos 2006 113 363 375 10.1111/j.2006.0030-1299.14714.x

[B21] Martiny AC Treseder K Pusch G Phylogenetic conservatism of functional traits in microorganisms ISME J 2013 7 830 838 10.1038/ismej.2012.160 23235290 PMC3603392

[B22] Faith DP Conservation evaluation and phylogenetic diversity Biol Conserv 1992 61 1 10 10.1016/0006-3207(92)91201-3

[B23] Cadotte MW Davies TJ Regetz J Kembel SW Cleland E Oakley TH Phylogenetic diversity metrics for ecological communities: integrating species richness, abundance, and evolutionary history Ecol Lett 2010 13 96 105 10.1111/j.1461-0248.2009.01405.x 19903196

[B24] Mooers AØ Heard SB Inferring evolutionary process from phylogenetic tree shape Q Rev Biol 1997 72 31 54 10.1086/419657

[B25] Simpson EH Meaasurement of diversity Nature 1949 163 688 10.1038/163688a0

[B26] Haegeman B Hamelin J Moriaty J Nael P Dushoff J Weitz JS Robust estimation of microbial diversity in theory and in practice ISME J 2013 doi:10.1038/ismej.2013.10 10.1038/ismej.2013.10 PMC3660670 23407313

[B27] Goltsman D Community Genomic, Proteomic, and Transcriptomic Analyses of Acid Mine Drainage Biofilm Communities PhD thesis 2013 Berkeley, California, USA: University of California Berkeley, Environmental Science, Policy and Management Department

[B28] Roberts A Pimentel H Trapnell C Pachter L Identification of novel transcripts in annotated genomes using RNA-Seq Bioinf 2011 27 2325 2329 10.1093/bioinformatics/btr355 21697122

[B29] Edgar RC Search and clustering orders of magnitude faster than BLAST Bioinf 2010 26 2460 2461 10.1093/bioinformatics/btq461 20709691

[B30] Emerson JB Thomas BC Andrade K Allen EE Heidelberg KB Banfield JF Dynamic viral populations in hypersaline systems as revealed by metagenomic assembly Appl Environ Microbiol 2012 78 6309 6320 10.1128/AEM.01212-12 22773627 PMC3416638

[B31] Emerson JB Andrade K Thomas BC Norman A Allen EE Heidelberg KB Banfield JF Virus-host and CRISPR dynamics in archaea-dominated Hypersaline Lake Tyrrell, Victoria, Australia Archaea 2013 2013 370871 23853523 10.1155/2013/370871 PMC3703381

[B32] Emerson JB Thomas BC Andrade K Heidelberg KB Banfield JF New approaches indicate constant viral diversity despite shifts in assemblage structure in an Australian hypersaline lake Appl Environ Microbiol In Press 10.1128/AEM.01946-13 PMC3811486 23995931

[B33] Margulies M Egholm M Altman WE Attiya S Bader JS Bemben LA Berka J Braverman MS Chen Y-J Chen Z Dewell SB Du L Fierro JM Gomes XV Godwin BC He W Helgesen S Ho CH Irzyk GP Jando SC Alenquer MLI Jarvie TP Jirage KB Kim J-B Knight JR Lanza JR Leamon JH Lefkowitz SM Lei M Li J Genome sequencing in microfabricated high-density picolitre reactors Nature 2005 437 376 380 16056220 10.1038/nature03959 PMC1464427

[B34] Simpson JT Wong K Jackman SD Schein JE Jones SJM Birol I ABySS: a parallel assembler for short read sequence data Genome Res 2009 19 1117 1123 10.1101/gr.089532.108 19251739 PMC2694472

[B35] Zerbino DR Birney E Velvet: algorithms for de novo short read assembly using de Bruijn graphs Genome Res 2008 18 821 829 10.1101/gr.074492.107 18349386 PMC2336801

[B36] Hyatt D Chen G-L LoCascio PF Land ML Larimer FW Hauser LJ Prodigal: prokaryotic gene recognition and translation initiation site identification BMC Bioinf 2010 11 119 10.1186/1471-2105-11-119 PMC2848648 20211023

[B37] Quevillon E Silventoinen V Pillai S Harte N Mulder N Apweiler R Lopez R InterProScan: protein domains identifier Nucleic Acids Res 2005 33 W116 W120 10.1093/nar/gki442 15980438 PMC1160203

[B38] Edgar RC MUSCLE: multiple sequence alignment with high accuracy and high throughput Nucleic Acids Res 2004 32 1792 1797 10.1093/nar/gkh340 15034147 PMC390337

[B39] Price MN Dehal PS Arkin AP FastTree: computing large minimum evolution trees with profiles instead of a distance matrix Mol Biol Evol 2009 26 1641 1650 10.1093/molbev/msp077 19377059 PMC2693737

[B40] Yelton AP Williams KH Fournelle J Wrighton KC Handley KM Banfield JF Vanadate and acetate biostimulation of contaminated sediments decreases diversity, selects for specific taxa, and decreases aqueous v(5+) concentration Environ Sci Technol 2013 47 6500 6509 23713472 10.1021/es4006674

[B41] Miller CS Baker BJ Thomas BC Singer SW Banfield JF EMIRGE: reconstruction of full-length ribosomal genes from microbial community short read sequencing data Genome Biol 2011 12 R44 10.1186/gb-2011-12-5-r44 21595876 PMC3219967

[B42] Miller CS Handley KM Wrighton KC Frischkorn KR Thomas BC Banfield JF Short-Read assembly of full-length 16S Amplicons reveals bacterial diversity in subsurface sediments PLoS ONE 2013 8 2 e56018 doi: 10.1371/journal.pone.0056018 10.1371/journal.pone.0056018 23405248 PMC3566076

[B43] Nawrocki EP Kolbe DL Eddy SR Infernal 1.0: inference of RNA alignments Bioinf 2009 25 1335 1337 10.1093/bioinformatics/btp157 PMC2732312 19307242

[B44] Price MN Dehal PS Arkin AP FastTree 2 – approximately maximum-likelihood trees for large alignments PLoS ONE 2010 5 e9490 Doi: 10.1371/journal.pone.0009490 10.1371/journal.pone.0009490 20224823 PMC2835736

[B45] Kerekes J Species Diversity, Ecology and Laccase Gene Diversity of Saprotrophic Fungi across Different Plant Community Types PhD thesis 2011 Berkeley, California, USA: University of California, Berkeley, Department of Plant and Microbial Biology

[B46] Amend AS Seifert K Samson R Bruns TD Indoor fungal composition is geographically patterned and more diverse in temperate zones than in the tropics Proc Natl Acad Sci USA 2010 107 13748 13753 10.1073/pnas.1000454107 20616017 PMC2922287

[B47] Tedersoo L Jairus T Horton BM Abarenkov K Suvi T Saar I Kõljalg U Strong host preference of ectomycorrhizal fungi in a Tasmanian wet sclerophyll forest as revealed by DNA barcoding and taxon-specific primers New Phytol 2008 180 479 490 10.1111/j.1469-8137.2008.02561.x 18631297

[B48] Caporaso JG Kuczynski J Stombaugh J Bittinger K Bushman FD Costello EK Fierer N Peña AG Goodrich JK Godron JI Huttley GA Kelley ST Knights D Koenig JE Ley RE Lozupone CA McDonald D Muegge BD Pirrung M Reeder J Sevinsky JR Turnbaugh PJ Walters WA Widmann J Yatsunenko T Zaneveld J Knight R QIIME allows analysis of high- throughput community sequencing data Nat Methods 2010 7 335 336 10.1038/nmeth.f.303 20383131 PMC3156573

[B49] Colless DH Review of Phylogenetics: the theory and practice of Phylogenetic systematics Syst Zool 1982 31 100 104 10.2307/2413420

[B50] Jaccard P Distribution de la flore alpine dans le basin de dranses et dans quelques regions voisines Bull Société Vaudoise Sci Natur 1901 37 241272

[B51] Lozupone C Knight R UniFrac: a new Phylogenetic method for comparing microbial communities Micrbiol 2005 71 8228 8235 10.1128/AEM.71.12.8228-8235.2005 PMC1317376 16332807

[B52] Ward JH Hierarchical Grouping to Optimize an Objective Function J Am Stat Assoc 1963 58 236 244 10.1080/01621459.1963.10500845

[B53] Sogin ML Morrison HG Huber JA Welch DM Huse SM Neal PR Arrieta JM Herndl GJ Microbial diversity in the deep sea and the underexplored “rare biodsphere Proc Natl Acad Sci USA 2006 103 12115 12120 10.1073/pnas.0605127103 16880384 PMC1524930

[B54] Hooper DU Vitousek PM The effects of plant composition and diversity on ecosystem processes Science 1997 277 1302 1305 10.1126/science.277.5330.1302

[B55] Tilman D Lehman CL Thomson KT Plant diversity and ecosystem productivity: theoretical considerations Proc Natl Acad Sci USA 1997 94 1857 1861 10.1073/pnas.94.5.1857 11038606 PMC20007

[B56] Silvertown J Plant coexistence and the niche Trends Ecol Evol 2004 19 605 611 10.1016/j.tree.2004.09.003

[B57] Ackerly DD Cornwell WK A trait-based approach to community assembly: partitioning of species trait values into within- and among-community components Ecol Lett 2007 10 135 145 10.1111/j.1461-0248.2006.01006.x 17257101

[B58] Chazdon RL Careaga S Webb C Vargas O Community and phylogenetic structure of reproductive traits of woody species in wet tropical forests Ecol Monogr 2003 73 331 348 10.1890/02-4037

[B59] Brumfield RT Tello JG Cheviron ZA Carling MD Crochet N Rosenberg KV Phylogenetic conservatism and antiquity of a tropical specialization: Army-ant-following in the typical antbirds (Thamnophilidae) Mol Phylogenet Evol 2007 45 1 13 10.1016/j.ympev.2007.07.019 17768072

[B60] Placella SA Brodie EL Firestone MK Rainfall-induced carbon dioxide pulses result from sequential resuscitation of phylogenetically clustered microbial groups Proc Natl Acad Sci USA 2012 109 10931 10936 10.1073/pnas.1204306109 22715291 PMC3390866

[B61] Langille MGI Zaneveld J Caporaso JG McDonald D Knights D Reyes JA Celemente JC Burkepile DE Vega Thurber RL Knight R Beiko RG Huttenhower C Predictive functional profiling of microbial communities using 16S rRNA marker gene sequences Nat Biotechnol 2013 31 814 821 10.1038/nbt.2676 23975157 PMC3819121

[B62] Galvão TC Mohn WW de Lorenzo V Exploring the microbial biodegradation and biotransformation gene pool Trends Biotechnol 2005 23 497 506 10.1016/j.tibtech.2005.08.002 16125262

[B63] Ferrer M Beloqui A Timmis KM Golyshin KN Metagenomics for mining new genetic resources of microbial communities J Mol Microbiol Biotechnol 2009 16 109 123 10.1159/000142898 18957866

[B64] Singh BK Macdonald CA Drug discovery from uncultivable microorganisms Drug Discov Today 2010 15 792 799 10.1016/j.drudis.2010.07.002 20656054

[B65] Blum MG François O Which random processes describe the tree of life? A large-scale study of phylogenetic tree imbalance Syst Biol 2006 55 685 691 10.1080/10635150600889625 16969944

[B66] Fisher RA Corbet AS Williams CB The relation between the number of species and the number of individuals in a random sample of an animal population J Anim Ecol 1943 12 42 58 10.2307/1411

[B67] Magurran AE Henderson PA Explaining the excess of rare species in natural species abundance distributions Nature 2003 422 714 716 10.1038/nature01547 12700760

[B68] Sunagawa S Woodley CM Medina M Threatened corals provide underexplored microbial habitats PLoS ONE 2010 5 e9554 Doi: 10.1371/journal.pone.0009554 10.1371/journal.pone.0009554 20221265 PMC2832684

