# Supplementary material for: Utilizing novel diversity estimators to quantify multiple dimensions of microbial biodiversity across domains
Source: BMC Microbiol. 2013 Nov 15;13:259. doi: 10.1186/1471-2180-13-259 (PMC3840555; doi:10.1186/1471-2180-13-259)
Supplement: Additional file 1: Table S1 — – Results of the community composition analyses (Jaccard and Unifrac) for the four environmental microbial community datasets. Figure S1. – Acid mine drainage bacteria and archaea (GAIIx) diversity profiles. Figure S2. – Hypersaline lake viruses methyltransferase diversity profiles. Figure S3. – Hypersaline lake viruses concanavalin A-like glucanases/lectins diversity profiles. Figure S4. – Substrate-associated soil fungi forest diversity profiles. Figure S5. – Acid mine drainage bacteria and archaea (HiSeq) phylogenetic (UniFrac) and taxonomic (Jaccard) hierarchical dissimilarity clusters. Figure S6. – Acid mine drainage bacteria and archaea (GAIIx) phylogenetic (UniFrac) and taxonomic (Jaccard) hierarchical dissimilarity clusters. Figure S7. – Hypersaline lake viruses Cluster 667 phylogenetic (UniFrac) and taxonomic (Jaccard) hierarchical dissimilarity clusters. Figure S8. – Hypersaline lake viruses methyltransferase phylogenetic (UniFrac) and taxonomic (Jaccard) hierarchical dissimilarity clusters. Figure S9. – Hypersaline lake viruses concanavalin A-like glucanases/lectins phylogenetic (UniFrac) and taxonomic (Jaccard) hierarchical dissimilarity clusters. Figure S10. – Subsurface bacteria phylogenetic (UniFrac) and taxonomic (Jaccard) hierarchical dissimilarity clusters. Figure S11. – Substrate-associated soil fungi phylogenetic (UniFrac) and taxonomic (Jaccard) hierarchical dissimilarity clusters. [file 1471-2180-13-259-S1.pdf]

Utilizing novel diversity estimators to quantify multiple dimensions of microbial biodiversity across domains

Hannah M. Doll[1*], David W. Armitage[2], Rebecca A. Daly[3,4,5], Joanne B. Emerson[6,7], Daniela S. Aliaga Goltsman[1,8], Alexis Yelton[1,9], Jennifer Kerekes[3], Mary K. Firestone[1,4] and Matthew D. Potts[1]

[1] Environmental Science, Policy, and Management, University of California, Berkeley, California 94720 USA

[2] Integrative Biology, University of California, Berkeley, California 94720 USA

[3] Plant and Microbial Biology, University of California, Berkeley, California 94720 USA

[4] Ecology Department, Earth Sciences Division, Lawrence Berkeley National Laboratory, Berkeley, California 94720 USA

[5] Current address: Department of Microbiology, The Ohio State University, Columbus, Ohio 43210 USA

[6] Earth and Planetary Science, University of California, Berkeley, California 94720 USA

[7] Current address: Cooperative Institute for Research in Environmental Sciences, University of Colorado, Boulder, Colorado 80309 USA

[8] Current address: Department of Microbiology and Immunology, School of Medicine, Stanford University, Stanford, California 94305 USA

[9] Current address: Civil and Environmental Engineering, Massachusetts Institute of Technology, Cambridge, Massachusetts 02139 USA

[*] Corresponding author: hdoll@berkeley.edu

Additional file 1

Additional results: Community dissimilarity comparisons

In order to compare the diversity calculations produced by diversity profiles to more traditional calculations of community composition for the same datasets, four different statistics of pairwise community dissimilarity were computed (abundance-weighted Jaccard, unweighted Jaccard, abundance-weighted UniFrac, and unweighted UniFrac). Please see the Methods section of the manuscript for further description of these indices.

Acid mine drainage bacteria and archaea:

Data from the HiSeq-platform showed very similar clustering topologies between Jaccard and UniFrac, as well as between abundance-weighted and unweighted samples (Additional file 1: Table S1, Figure S5). In the weighted calculations, the bioreactor samples, which were taken from the same reactor at different time points, matched more closely with each other than with environmental samples. However, samples clustered rather randomly in the unweighted calculations. The topologies of hierarchical clustering results were also similar between the Jaccard and UniFrac methods for both abundance-weighted and unweighted GAIIx samples: The bioreactor samples cluster together in weighted calculations (Additional file 1: Figure S6).

Hypersaline lake viruses:

The relative topological differences between community dissimilarity measures (UniFrac and Jaccard) for the hypersaline lake viruses dataset were greater between abundance-weighted and unweighted samples of the same type of information (phylogenetic and taxonomic) than between the two metrics under the same abundance-weighting assumption (Additional file 1: Table S1, Figures S7, S8, S9).

Subsurface bacteria:

Hierarchical clustering of phylogenetic (UniFrac) and taxonomic (Jaccard) community dissimilarity indices gave similar topologies (Additional file 1: Table S1, Figure S10). However, these topologies differed between abundance-weighted and presence/absence formulations. In the latter, the background and acetate samples were most similar. When weighted by sequence abundances the vanadium plus acetate treatments clustered more closely with acetate-only treatments.

Substrate-associated soil fungi:

The topology of hierarchical dissimilarity clusters were most similar between unweighted Jaccard and UniFrac methods (Additional file 1: Table S1, Figure S11). The abundance-weighted variants of these methods arrived at slightly different topologies. However, both weighted and unweighted analyses grouped samples from similar habitats together, though the clustering of substrates or time points for each habitat varied with the method used.

Table S1 – Results of the community composition analyses (Jaccard and Unifrac) for the four environmental microbial community datasets.

<table>
<tr><th></th><th>Naïve Composition Results</th><th>Was This Predicted?</th><th>Similarity Composition Results</th><th>Was This Predicted?</th></tr>
<tr><td>Acid mine drainage bacteria and archaea</td><td>Bioreactors cluster separately from environmental samples</td><td>No</td><td>Bioreactors cluster separately from environmental samples</td><td>Yes</td></tr>
<tr><td>Hypersaline lake viruses</td><td>Clusters by site then year</td><td>Yes</td><td>Clusters by site then year</td><td>Yes</td></tr>
<tr><td>Subsurface bacteria</td><td>Clusters reflect treatments</td><td>Yes</td><td>Clusters reflect treatments</td><td>Yes</td></tr>
<tr><td rowspan="3">Substrate-associated soil fungi</td><td>Unifrac and Jaccard mostly cluster first by Community</td><td>Yes</td><td>Unifrac clusters by Substrate+Community, then by Community</td><td>Yes</td></tr>
<tr><td>They then cluster alternatively by either Timepoint or Substrate</td><td>No</td><td rowspan="2">Jaccard mostly clusters like Unifrac, except Forest samples cluster first by Community+Timepoint (not substrate)</td><td rowspan="2">No</td></tr>
<tr><td>Straw in Grassland T2 and Straw in Forest T2 break the above trends and cluster together in both Unifrac and Jaccard</td><td>No</td></tr>
</table>

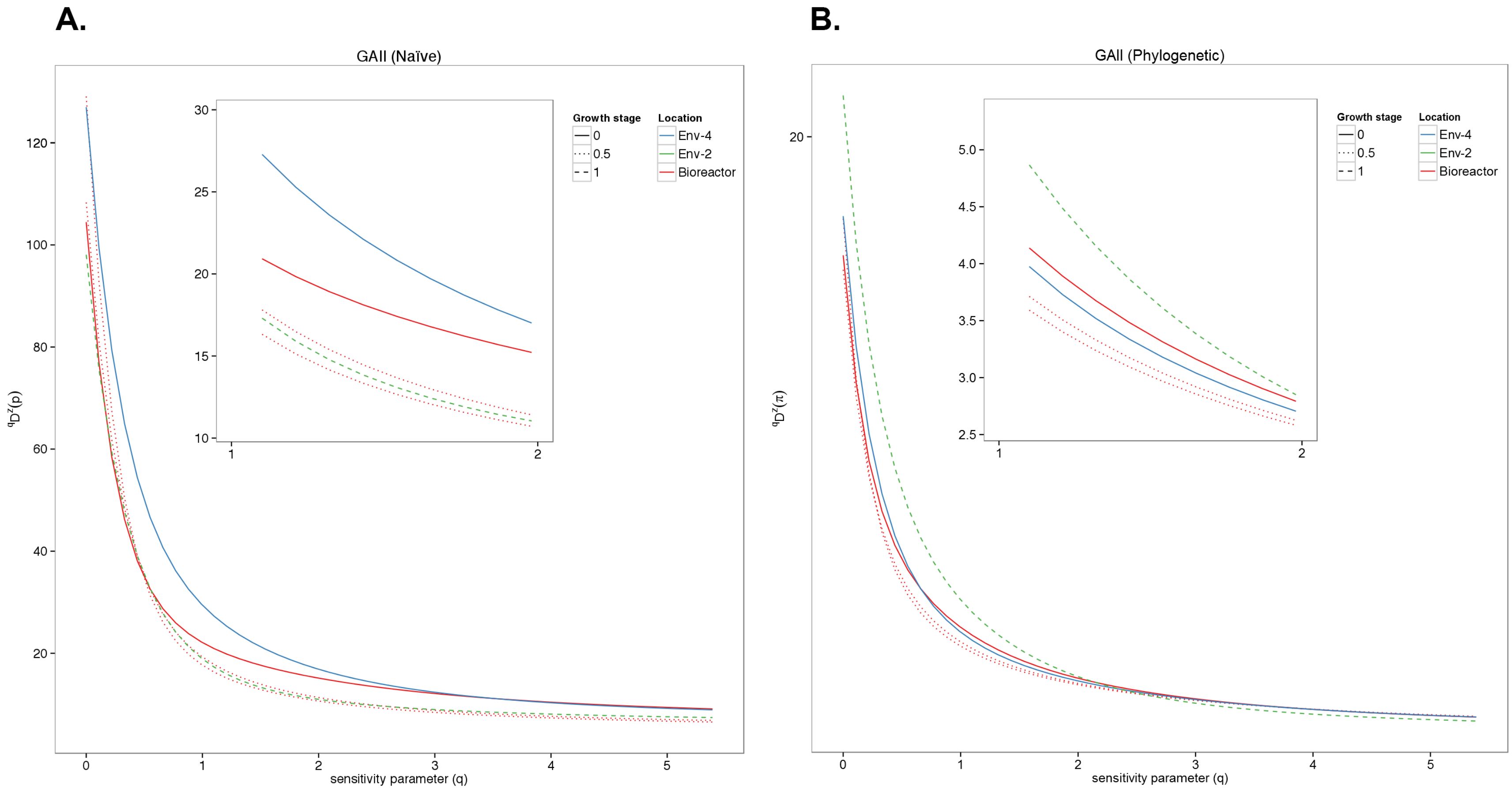

Figure S1 – Acid mine drainage bacteria and archaea (GAIIx) diversity profiles.

(A) Naïve and (B) similarity-based (phylogenetic relatedness) diversity profiles calculated from the acid mine drainage bacteria and archaea GAIIx data.

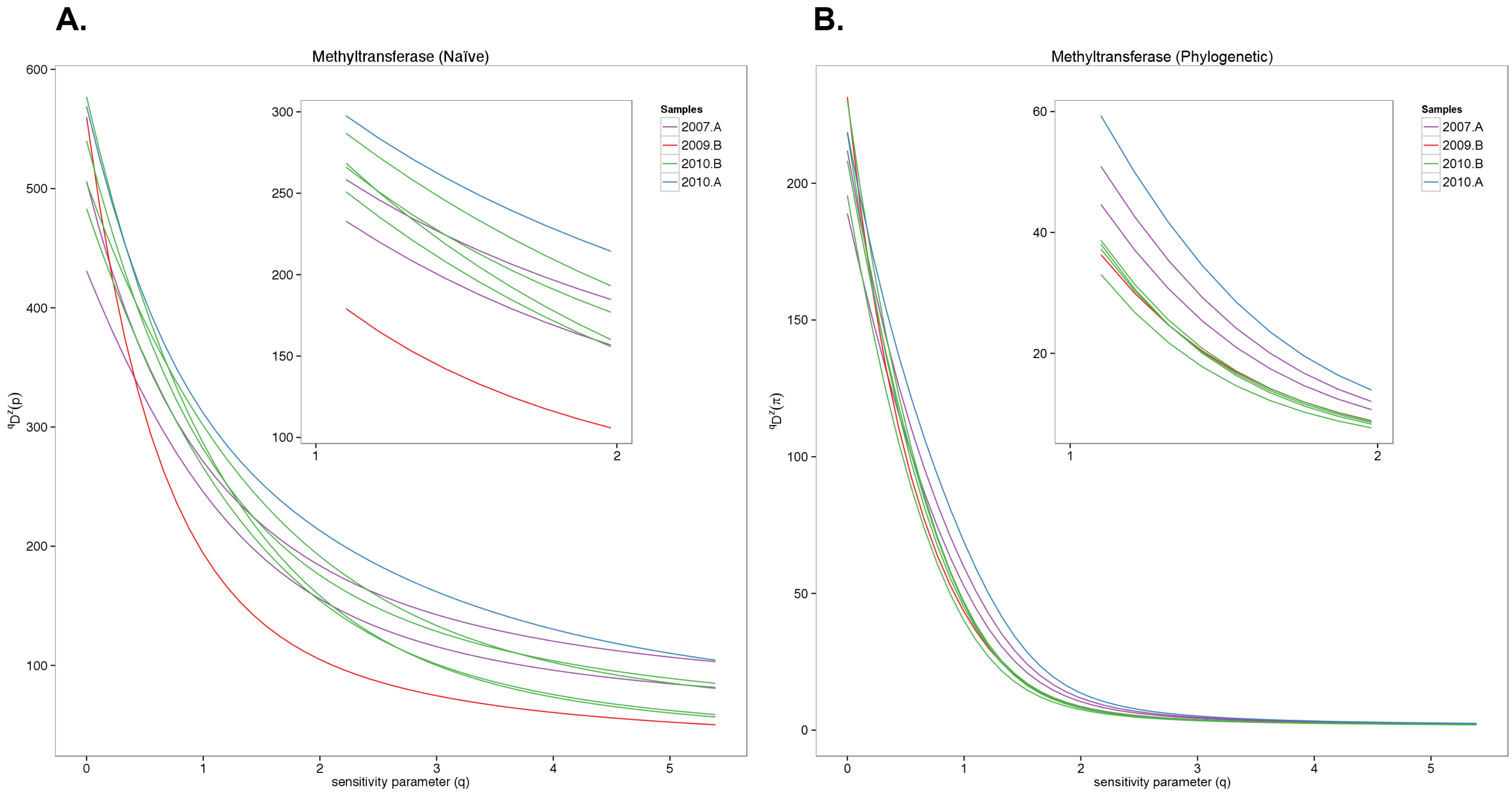

Figure S2 – Hypersaline lake viruses methyltransferase diversity profiles.

(A) Naïve and (B) similarity-based (phylogenetic relatedness) diversity profiles calculated from the hypersaline lake viruses methyltransferase data.

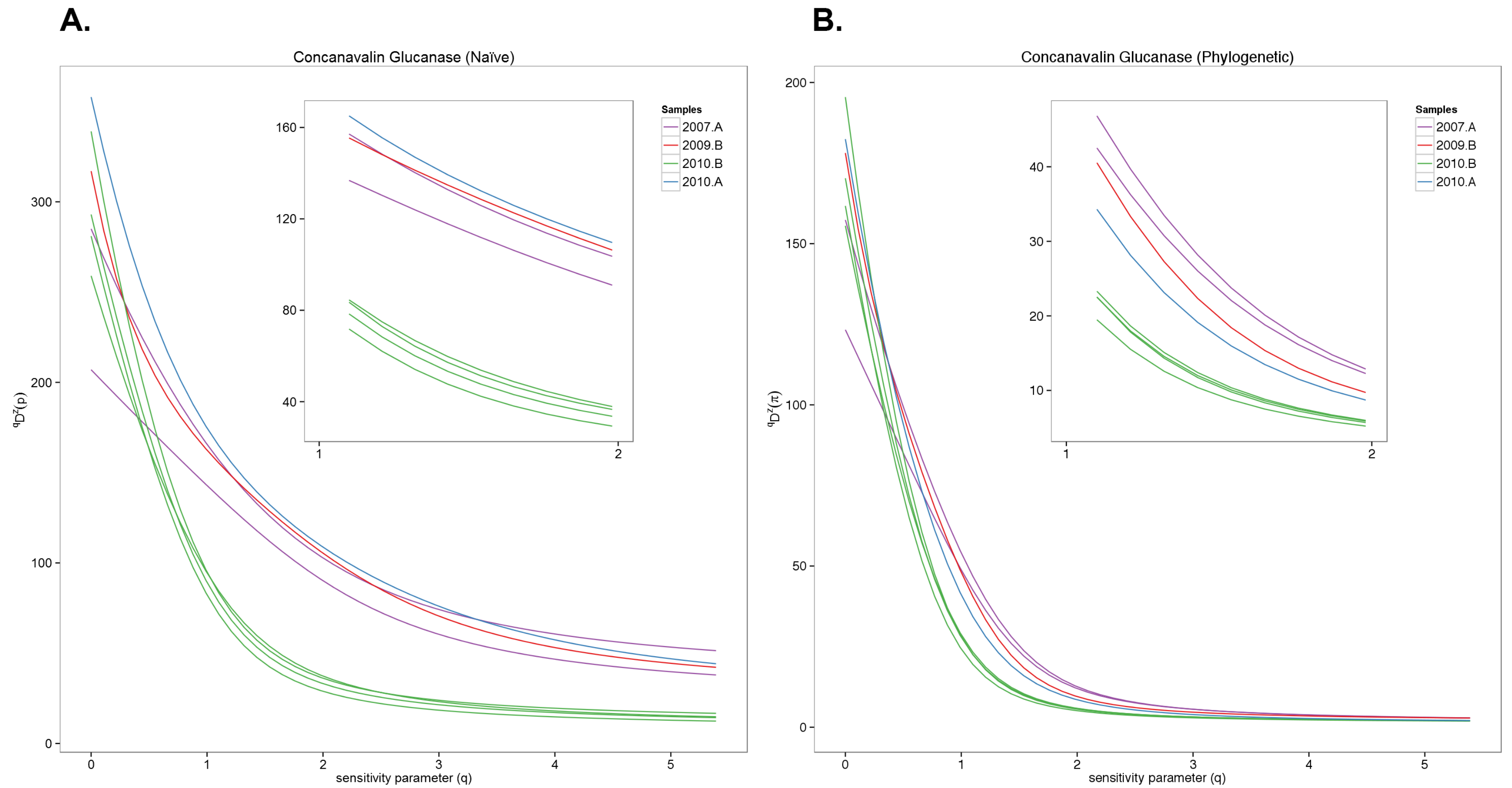

Figure S3 – Hypersaline lake viruses concanavalin A-like glucanases/lectins diversity profiles.

(A) Naïve and (B) similarity-based (phylogenetic relatedness) diversity profiles calculated from the hypersaline lake viruses concanavalin A-like glucanases/lectins data.

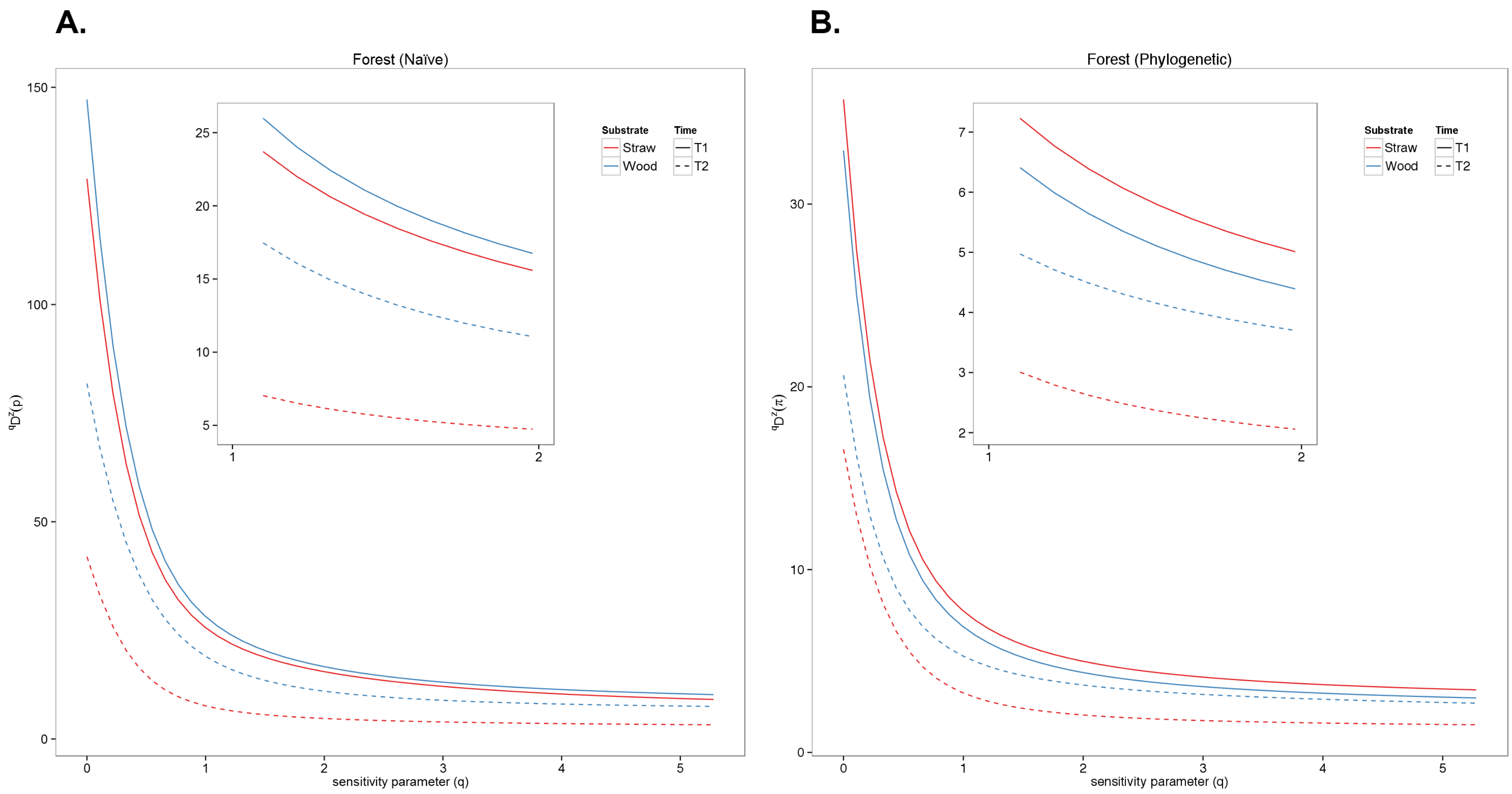

Figure S4 – Substrate-associated soil fungi forest diversity profiles.

(A) Naïve and (B) similarity-based (phylogenetic relatedness) diversity profiles calculated from the substrate-associated soil fungi forest data.

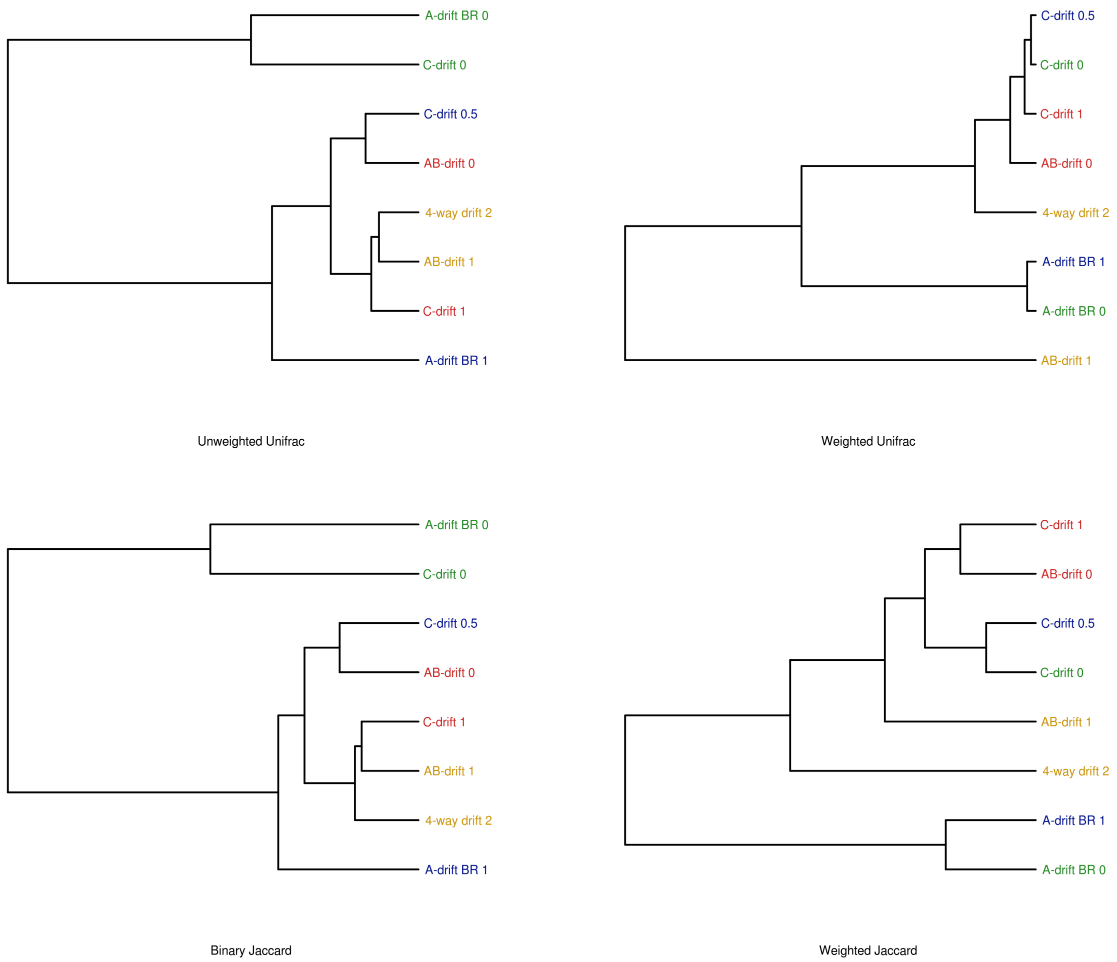

Figure S5 – Acid mine drainage bacteria and archaea (HiSeq) phylogenetic (UniFrac) and taxonomic (Jaccard) hierarchical dissimilarity clusters.

(Top Left) Unweighted Unifrac, (Top Right) abundance-weighted Unifrac, (Bottom Left) unweighted Jaccard, and (Bottom Right) abundance-weighted Jaccard community composition dendrograms calculated from the acid mine drainage bacteria and archaea HiSeq data.

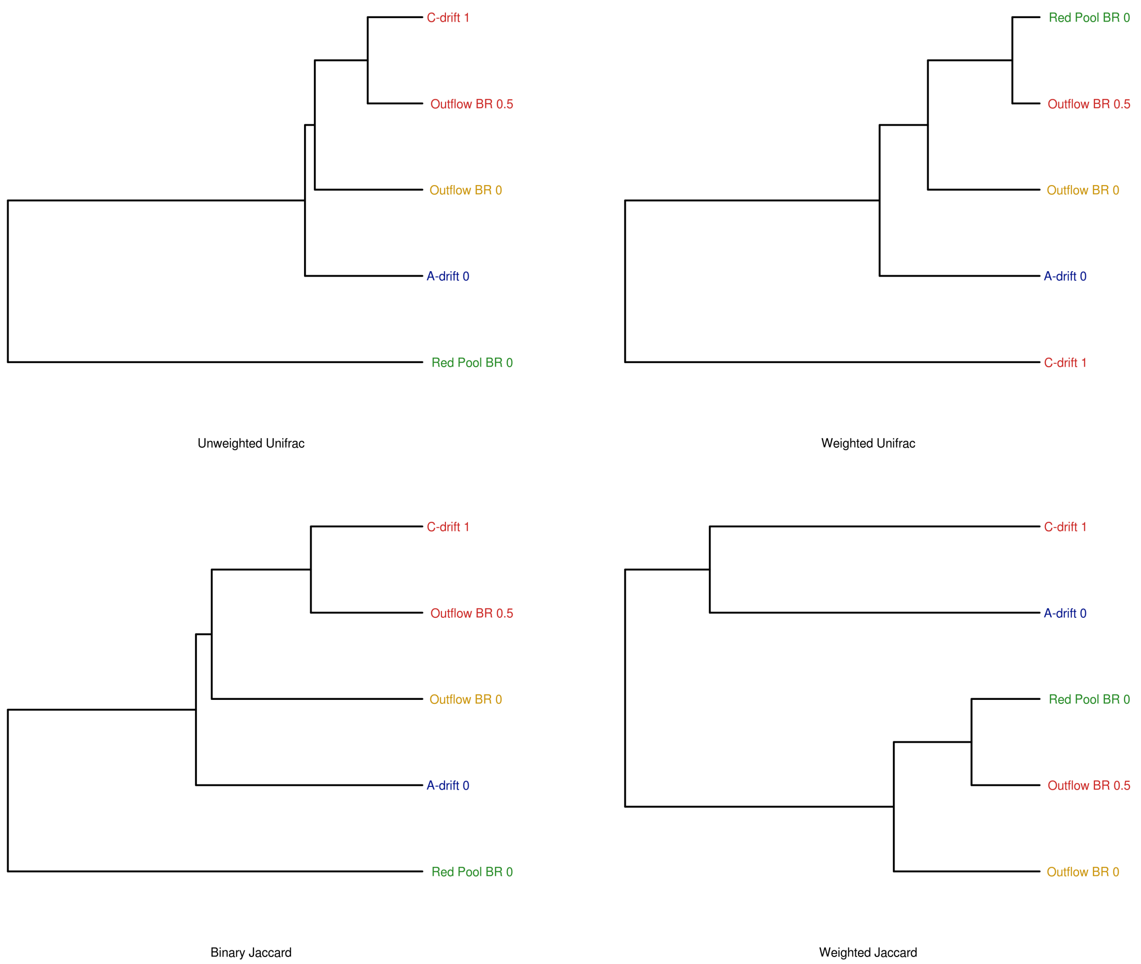

Figure S6 – Acid mine drainage bacteria and archaea (GAIIx) phylogenetic (UniFrac) and taxonomic (Jaccard) hierarchical dissimilarity clusters.

(Top Left) Unweighted Unifrac, (Top Right) abundance-weighted Unifrac, (Bottom Left) unweighted Jaccard, and (Bottom Right) abundance-weighted Jaccard community composition dendrograms calculated from the acid mine drainage bacteria and archaea GAIIx data.

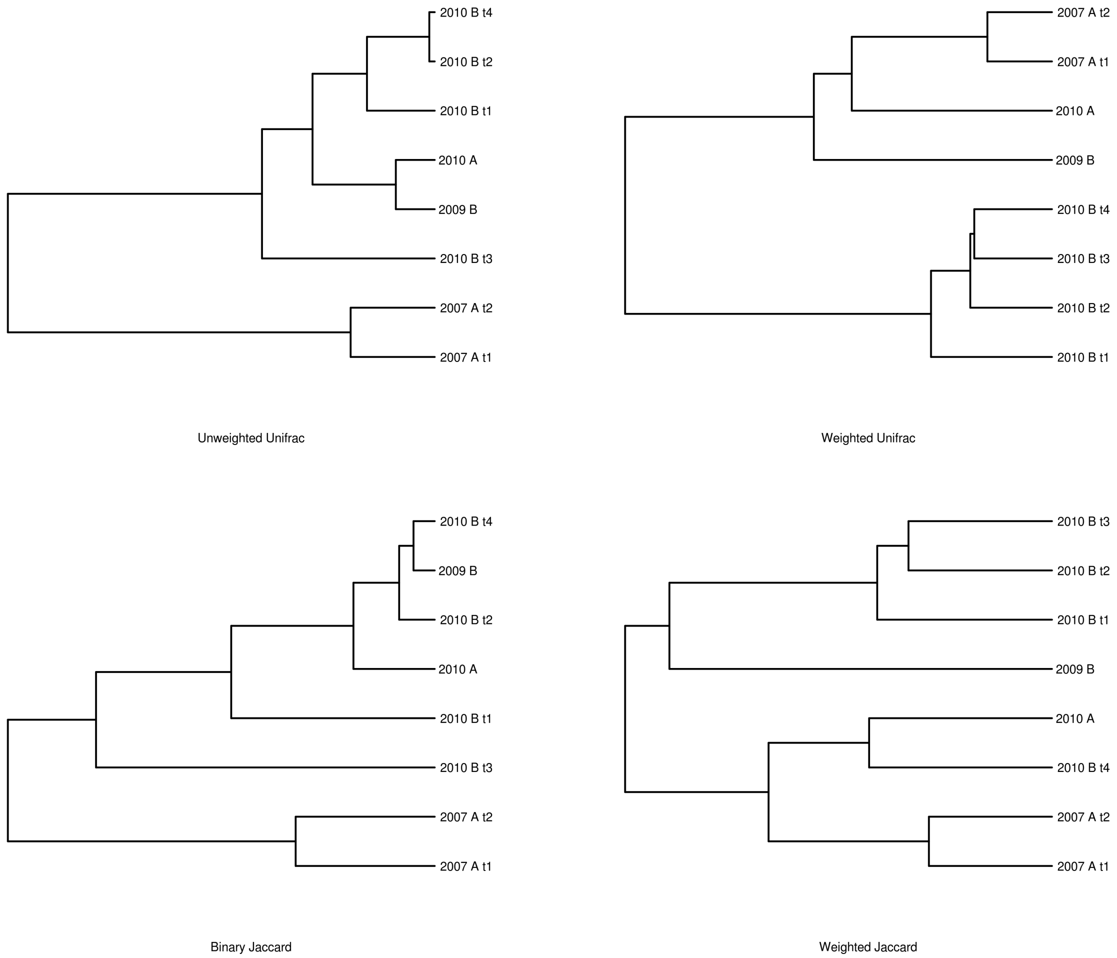

Figure S7 – Hypersaline lake viruses Cluster 667 phylogenetic (UniFrac) and taxonomic (Jaccard) hierarchical dissimilarity clusters.

(Top Left) Unweighted Unifrac, (Top Right) abundance-weighted Unifrac, (Bottom Left) unweighted Jaccard, and (Bottom Right) abundance-weighted Jaccard community composition dendrograms calculated from the hypersaline lake viruses Cluster 667 data.

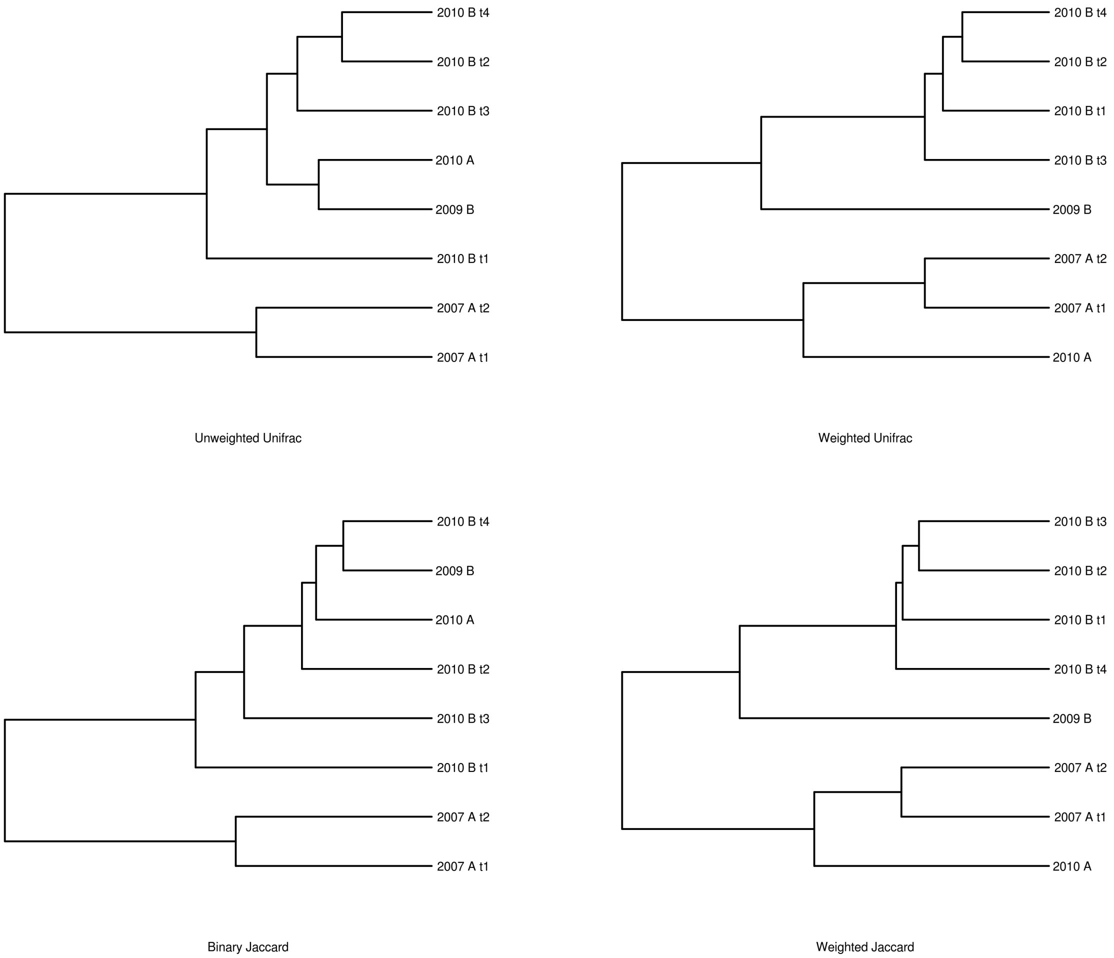

Figure S8 – Hypersaline lake viruses methyltransferase phylogenetic (UniFrac) and taxonomic (Jaccard) hierarchical dissimilarity clusters.

(Top Left) Unweighted Unifrac, (Top Right) abundance-weighted Unifrac, (Bottom Left) unweighted Jaccard, and (Bottom Right) abundance-weighted Jaccard community composition dendrograms calculated from the hypersaline lake viruses methyltransferase data.

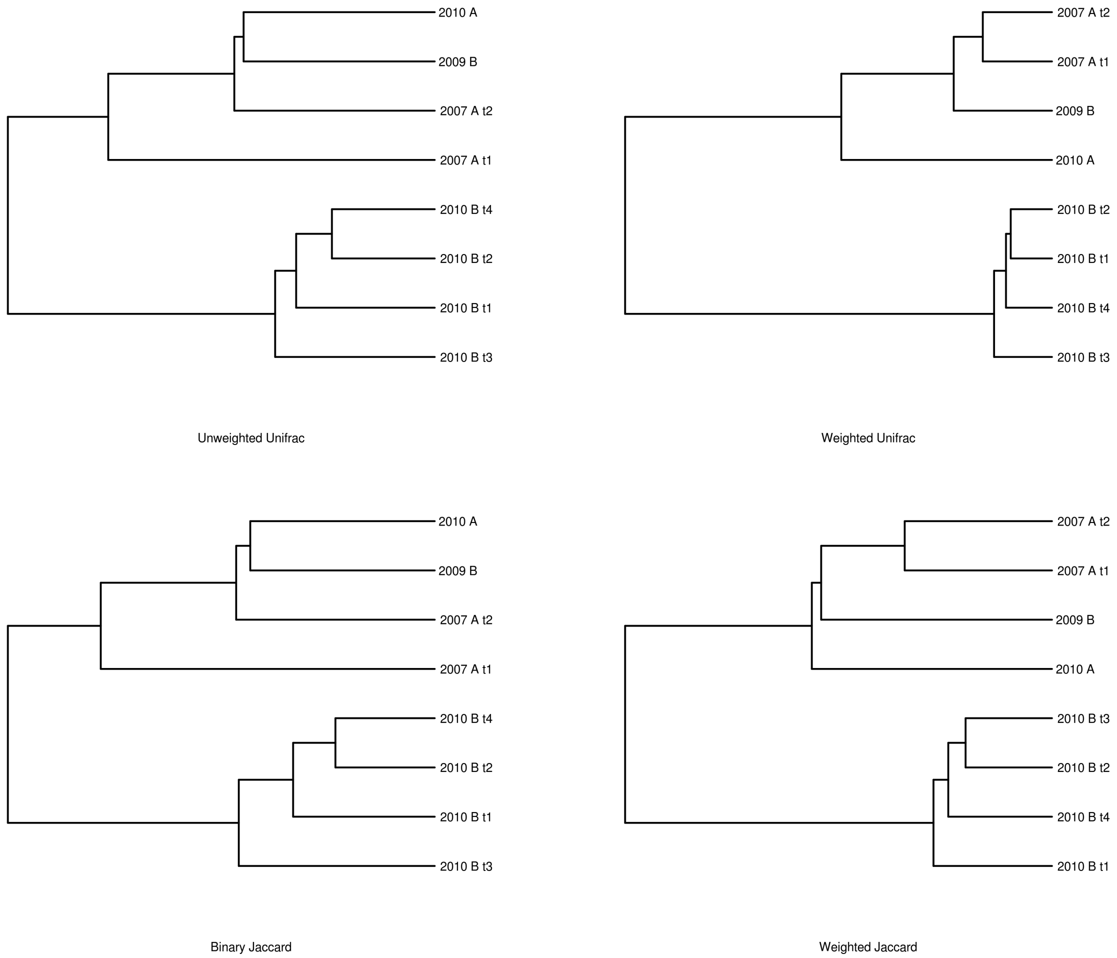

Figure S9 – Hypersaline lake viruses concanavalin A-like glucanases/lectins phylogenetic (UniFrac) and taxonomic (Jaccard) hierarchical dissimilarity clusters.

(Top Left) Unweighted Unifrac, (Top Right) abundance-weighted Unifrac, (Bottom Left) unweighted Jaccard, and (Bottom Right) abundance-weighted Jaccard community composition dendrograms calculated from the hypersaline lake viruses concanavalin A-like glucanases/lectins data.

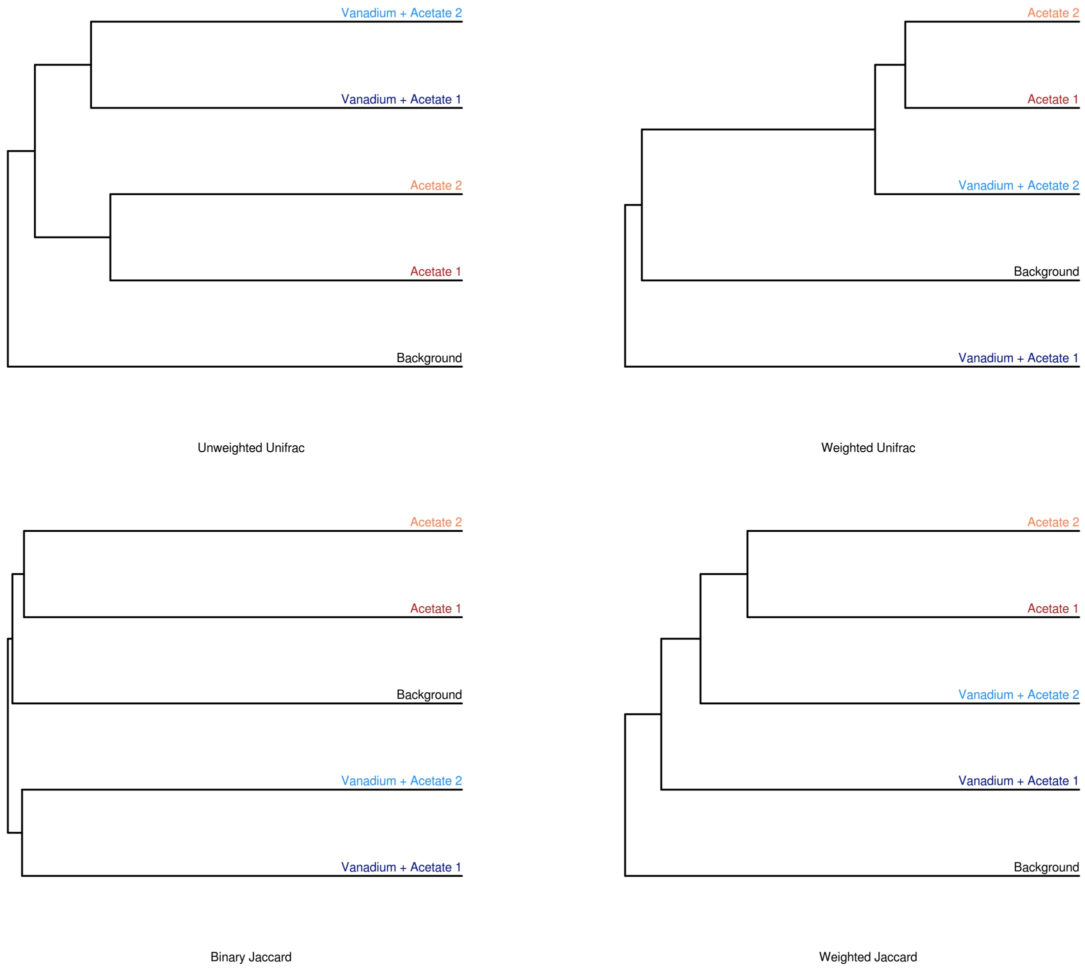

Figure S10 – Subsurface bacteria phylogenetic (UniFrac) and taxonomic (Jaccard) hierarchical dissimilarity clusters.

(Top Left) Unweighted Unifrac, (Top Right) abundance-weighted Unifrac, (Bottom Left) unweighted Jaccard, and (Bottom Right) abundance-weighted Jaccard community composition dendrograms calculated from the subsurface bacteria dataset.

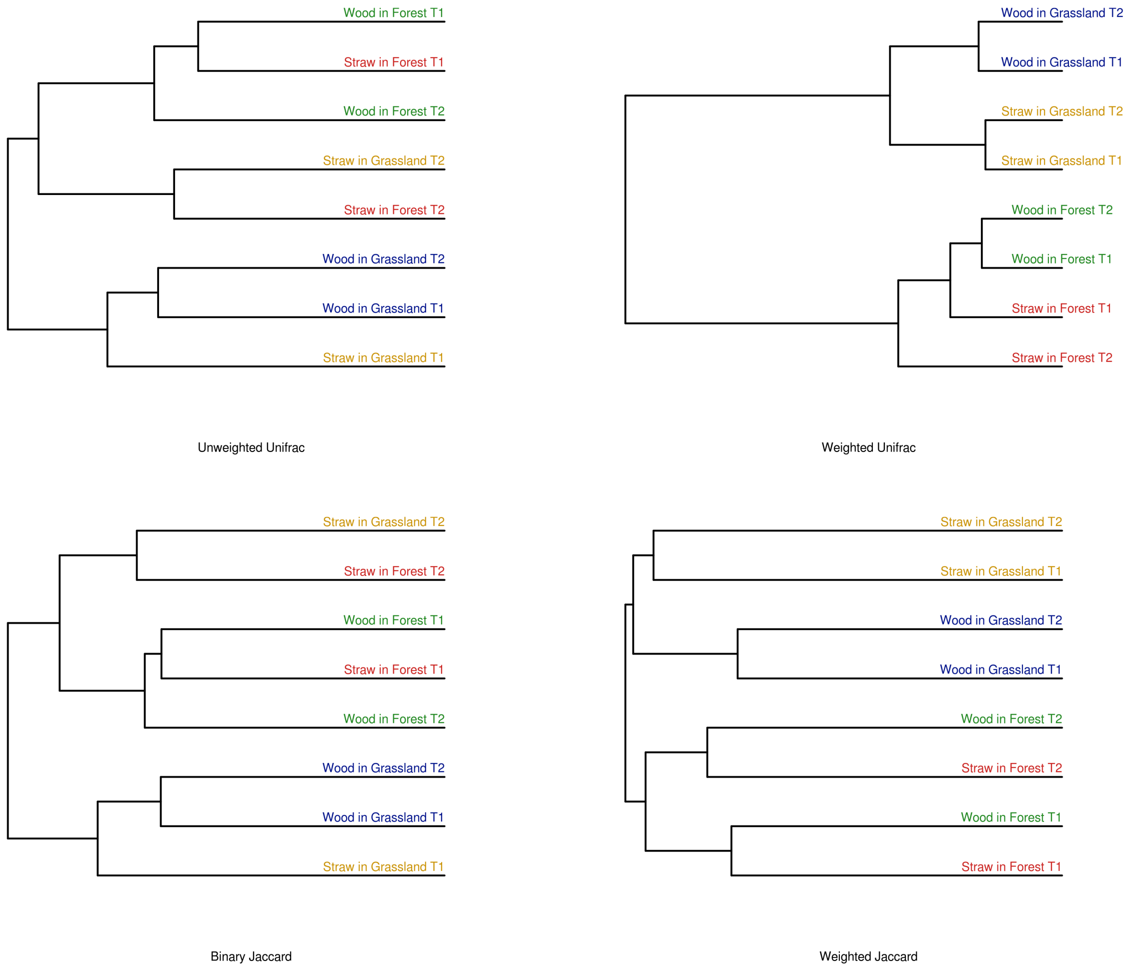

Figure S11 – Substrate-associated soil fungi phylogenetic (UniFrac) and taxonomic (Jaccard) hierarchical dissimilarity clusters.

(Top Left) Unweighted Unifrac, (Top Right) abundance-weighted Unifrac, (Bottom Left) unweighted Jaccard, and (Bottom Right) abundance-weighted Jaccard community composition dendrograms calculated from the substrate-associated soil fungi dataset.
